# Haplotype inference from unphased SNP data in heterozygous polyploids based on SAT

**DOI:** 10.1186/1471-2164-9-356

**Published:** 2008-07-30

**Authors:** Jost Neigenfind, Gabor Gyetvai, Rico Basekow, Svenja Diehl, Ute Achenbach, Christiane Gebhardt, Joachim Selbig, Birgit Kersten

**Affiliations:** 1 Bioinformatics, GabiPD team, Max Planck Institute of Molecular Plant Physiology, 14424 Potsdam-Golm, Germany; 2 Bioinformatics, Former RZPD German Resource Center for Genome Research GmbH, Heubnerweg 6, D-14059, Berlin, Germany; 3 Max Planck Institute for Plant Breeding Research, Carl von Linnè Weg 10, 50829 Köln, Germany; 4 Institute of Biochemistry and Biology, University of Potsdam, c/o MPI-MP, 14424 Potsdam, Germany

## Abstract

**Background:**

Haplotype inference based on unphased SNP markers is an important task in population genetics. Although there are different approaches to the inference of haplotypes in diploid species, the existing software is not suitable for inferring haplotypes from unphased SNP data in polyploid species, such as the cultivated potato (*Solanum tuberosum*). Potato species are tetraploid and highly heterozygous.

**Results:**

Here we present the software SATlotyper which is able to handle polyploid and polyallelic data. SATlo-typer uses the Boolean satisfiability problem to formulate Haplotype Inference by Pure Parsimony. The software excludes existing haplotype inferences, thus allowing for calculation of alternative inferences. As it is not known which of the multiple haplotype inferences are best supported by the given unphased data set, we use a bootstrapping procedure that allows for scoring of alternative inferences. Finally, by means of the bootstrapping scores, it is possible to optimise the phased genotypes belonging to a given haplotype inference. The program is evaluated with simulated and experimental SNP data generated for heterozygous tetraploid populations of potato. We show that, instead of taking the first haplotype inference reported by the program, we can significantly improve the quality of the final result by applying additional methods that include scoring of the alternative haplotype inferences and genotype optimisation. For a sub-population of nineteen individuals, the predicted results computed by SATlotyper were directly compared with results obtained by experimental haplotype inference via sequencing of cloned amplicons. Prediction and experiment gave similar results regarding the inferred haplotypes and phased genotypes.

**Conclusion:**

Our results suggest that Haplotype Inference by Pure Parsimony can be solved efficiently by the SAT approach, even for data sets of unphased SNP from heterozygous polyploids. SATlotyper is freeware and is distributed as a Java JAR file. The software can be downloaded from the webpage of the GABI Primary Database at . The application of SATlotyper will provide haplotype information, which can be used in haplotype association mapping studies of polyploid plants.

## Background

In the case of homozygous genotypes, such as maize or many other inbreeding crop species, haplotypes can be directly drawn from comparison of the amplified genomic sequence at a given locus between different individuals [1]. Difficulties arise if homozygous genotypes are not available, for example, in non-inbred, tetraploid potato [2]. In such cases, it is necessary to determine the haplotype phase from unphased SNP (single nucleotide polymorphism) data. There are several approaches for inferring haplotypes, based on (i) statistical methods, such as the EM algorithm and Gibbs sampling or (ii) the parsimony principle [3]. These approaches have, however, been developed for biallelic and diploid species. There is currently no software available for haplotype identification in more complex polyploids [2,4]. In the case of autotetraploids [4], one has to tackle more phase-unknown alleles than in diploids, which results in a combinatorial explosion of possible haplotypes.

In this study, we aimed at the development and evaluation of a generalised approach for calculating haplo-types in polyploid species using the parsimony principle. The goal of haplotype inference is to find a set of haplotypes explaining every genotype present in a given unphased population. The parsimony principle can be used to find the smallest set of haplotypes, such that each genotype in the population can be explained by a ploidy-specific number of haplotypes from the set of haplotypes. The objective of minimising the number of haplotypes explaining a SNP data set is called Haplotype Inference by Pure Parsimony (HIPP) [5] and was shown to be NP-hard [6]. Lynce and Marques-Silva recently formulated the problem as an instance of the Boolean satisfiability problem, called SAT [7,8], that can be solved orders of magnitude faster than the existing ILP (integer linear programming) formulation [7,9,10]. Unfortunately, the SAT formulation is also restricted to unphased biallelic SNP data of diploid species. Here, we present a generalisation for polyploids of the SAT approach developed by Lynce and Marques-Silva [7,8]. This generalisation resulted in the development of the SATlotyper software tool. We tested and evaluated SATlotyper with simulated and experimental data sets of unphased SNP sites from a specific potato locus. SNP data were obtained from different populations of tetraploid individuals. For a subset of individuals, we compared the computed haplotypes with experimental haplotypes identified by amplicon cloning and sequencing [2].

## Implementation

First, basic terms are defined and the basic problem is formulated. Then, the SAT model for biallelic polyploids is presented. This is followed by the extension of the model to polyploid and polyallelic SNP sites. After that, constraints are given for breaking symmetries in haplotypes and genotypes. Constraints are also formulated for alternative most parsimonious sets of explaining haplotypes as well as for alternative inferences of genotypes. A bootstrapping procedure for scoring haplotypes and a method for optimising alternative genotype inferences based on these scores is presented. Afterwards, lower and upper bounds of the most parsimonious explanation are mentioned and the definition of a norm for comparing genotypes is given. Finally, the realisation of SATlotyper is considered.

### Basic definitions

**Figure 1:**
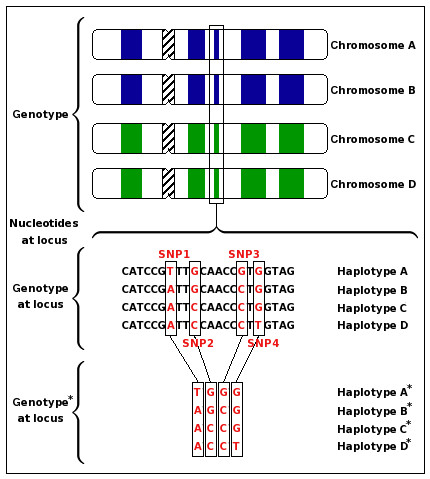
**Schema of terms related to the HIPP**. The total genotype of a tetraploid species consists of four chromosomes (Chromosome A-D) where two chromosomes come from the first parent (blue) and the other two from the second (green). A locus is a fixed position on a chromosome often consisting of many nucleotides. Four haplotypes (Haplotype A-D) represent the nucleotide sequence of the corresponding locus on the four chromosomes. The four haplotypes A*-D* represent the conflated data of genotype* and thus explain genotype* (see also Figure 2, Figure 3 and Figure 7). The example illustrates a population of one individual. A homozygous site of the presented individual and locus becomes a SNP site, if a second individual is added which is heterozygous at a site different from SNP1-4 (and vice versa).

The genome of every higher developed species, whether animal, plant or fungus, is based on a species-dependent number of homologous sets of chromosomes. The number of sets ranges from at least one set as it is found in yeast, which is a *haploid *species, followed by two sets in human (*diploid*) to much bigger numbers like four sets (*tetraploid*) in some varieties of the potato (e.g. *Solanum tuberosum*) or six homologous sets (*hexaploid*) in wheat (e.g. *Triticum aestivum*). The strawberry (e.g. *Fragaria ananassa*) can even have eight sets (*octaploid*). Chromosomes are sequences over the nucleotide alphabet, where the position of a specific nucleotide on the chromosome is called *site *or *locus *(Figure 1).

*Single Nucleotide Polymorphism *or *SNP *is a DNA sequence variation, occurring when a single nucleotide is altered [7]. Thus, a site in a population of a species is a SNP site if at least a second sort of nucleotide occurs at this site at least once.

An *allele *is a different form of some segment of a chromosome, such as a second sort of nucleotide at a SNP site. Here, we focus on SNP sites. A SNP site that contains two different alleles is called *biallelic*, a SNP site that contains three different alleles is called *triallelic *and a SNP site that contains four different alleles is called *tetraallelic*.

A *haplotype *is the genetic constitution of a sequence of nucleotides [7]. The underlying data that forms a haplotype can be the full DNA sequence in the region, or more commonly the SNP sites in that region [7]. Polyploid organisms contain two or more homologous haplotypes.

A *genotype *describes the conflated data of a set of homologous haplotypes. In other words, an *explanation *for a genotype is a ploidy-specific number of homologous haplotypes. An *unphased genotype *is a genotype for which no set of explaining haplotypes is defined. There are, however, many possible sets of haplotypes explaining one given unphased genotype. A *phased genotype *is a genotype for which at least one set of explaining haplotypes is defined. If for a given site all explaining haplotypes have the same value, then the genotype is said to be *homozygous *at that side. Otherwise the genotype is said to be *heterozygous *at that side.

Figure 1 illustrates how the different terms are related to each other.

### Problem formulation

A SNP site of an individual is a string over the nucleotide alphabet Σ = {A, C, G, T} with size determined by the ploidy of the considered species. A sequence of such SNP sites defines a genotype, with the number of SNP sites as the length of the genotype. Let *n *denote the number of individuals in the sample, *m *be the number of SNP sites, and *p *be the ploidy of the considered species. Furthermore, a specific genotype is denoted by *g*_*i*_, with 1 ≤ *i *≤ *n*, and for a specific site *j*, with 1 ≤ *j *≤ *m*, in *g*_*i *_we use *g*_*i*, *j*_. Finally, let $g_{i,j}^{l}$ with 1 ≤ *l *≤ *p*, denote the *l*^*th *^state at site *j *in genotype *i*. Given a set G of *n *genotypes, each of length *m*, the haplotype inference problem is that of finding a set H of not necessarily distinct haplotypes. Furthermore, for each genotype *g*_*i *_∈ G there is at least one set of *p *haplotypes {*h*^1^, ..., *h*^*p*^} ∈ H such that *g*_*i *_is explained by {*h*^1^, ... *h*^*p*^}. The values of nucleotides are determined by the number of different alleles at the corresponding SNP site: for a biallelic SNP site the values are {0, 1}, for a triallelic SNP site the values are {0, 1, 2}, while for a tetraallelic SNP site the values are {0, 1, 2, 3}. Thus, a specific haplotype *h*_*k *_is a string over the alphabet {0, 1, 2, 3}, with 1 ≤ *k *≤ |H|.

Polyploid genotypes are represented by sequences of *m *vectors, where the vectors encode the SNP sites of the given individual. The vectors are of size *p *and contain alphabetically sorted elements of the alphabet defined by the corresponding SNP site (SNP site always refers to a whole population). For instance, a tetraallelic SNP site of a tetraploid individual (*p *= 4) which is homozygous is encoded as a vector (0, 0, 0, 0), (1, 1, 1, 1), (2, 2, 2, 2) or (3, 3, 3, 3) depending on the allele found at the given site of the individual. A tetraallelic SNP site of a tetraploid individual at which two alleles occur twice is encoded as a vector (0, 0, 1, 1), (0, 0, 2, 2), (0, 0, 3, 3), (1, 1, 2, 2), (1, 1, 3, 3) or (2, 2, 3, 3) (in general there are eighteen possible encodings, see Table 1).

**Table 1:** Possible encodings of SNP sites

Number of alleles at given site	Possible encodings		
	
	Tetraallelic SNP site	Triallelic SNP site	Biallelic SNP site
Homozygous individual	(0, 0, 0, 0), (1, 1, 1, 1), (2, 2, 2, 2), (3, 3, 3, 3)	(0, 0, 0, 0), (1, 1, 1, 1), (2, 2, 2, 2)	(0, 0, 0, 0), (1, 1, 1, 1)
Biallelic individual	(0, 1, 1, 1), (0, 0, 1, 1), (0, 0, 0, 1), (0, 2, 2, 2), (0, 0, 2, 2), (0, 0, 0, 2), (0, 3, 3, 3), (0, 0, 3, 3), (0, 0, 0, 3), (1, 2, 2, 2), (1, 1, 2, 2), (1, 1, 1, 2), (1, 3, 3, 3), (1, 1, 3, 3), (1, 1, 1, 3), (2, 3, 3, 3), (2, 2, 3, 3), (2, 2, 2, 3)	(0, 1, 1, 1), (0, 0, 1, 1), (0, 0, 0, 1), (0, 2, 2, 2), (0, 0, 2, 2), (0, 0, 0, 2), (1, 2, 2, 2), (1, 1, 2, 2), (1, 1, 1, 2)	(0, 1, 1, 1), (0, 0, 1, 1), (0, 0, 0, 1)
Triallelic individual	(0, 1, 2, 2), (0, 1, 1, 2), (0, 0, 1, 2), (0, 1, 3, 3), (0, 1, 1, 3), (0, 0, 1, 3), (0, 2, 3, 3), (0, 2, 2, 3), (0, 0, 2, 3), (1, 2, 3, 3), (1, 2, 2, 3), (1, 1, 2, 3)	(0, 1, 2, 2), (0, 1, 1, 2), (0, 0, 1, 2)	
Tetraallelic individual	(0, 1, 2, 3)		

In this table all possible allele compositions for SNP sites in a tetraploid species are given. At tetraallelic SNP sites homozygous, biallelic, triallelic and tetraallelic individuals are possible. In contrast, at triallelic SNP sites only homozygous, biallelic and triallelic individuals are possible. Additionally, encodings that contain a fourth allele are not possible anymore. The number of possible allele compositions at biallelic SNP sites decreases analogously.

Note that the presented encodings hold only for tetraploid species. In general, the number of possible encodings increases exponentially with increasing ploidy. The increase is driven by all possible partitions of the ploidy using four summands for a tetraallelic individual, three summands for a triallelic individual and two summands for a biallelic individual. For instance, there are 2 different partitions for a biallelic site of a tetraploid individual: 1 + 3 = 4 and 2 + 2 = 4. Then there are 2! = 2 different encodings for the first partition and $\frac{2!}{2!}$ = 1 different encodings for the second partition. This makes 3 different encodings in total if this SNP site is biallelic (second row, third column). If the SNP site is triallelic, the number of encodings is multiplied by the number of two alleles chosen from three possible alleles: 3·$\left(\begin{array}{c} 3\\ 2 \end{array}\right)$ = 9 (second row, second 2 column). If the SNP site is tetraallelic, the number of encodings is multiplied by the number of two alleles chosen from four possible alleles: 3·$\left(\begin{array}{c} 4\\ 2 \end{array}\right)$ = 18 (second row, first column).

**Figure 2:**
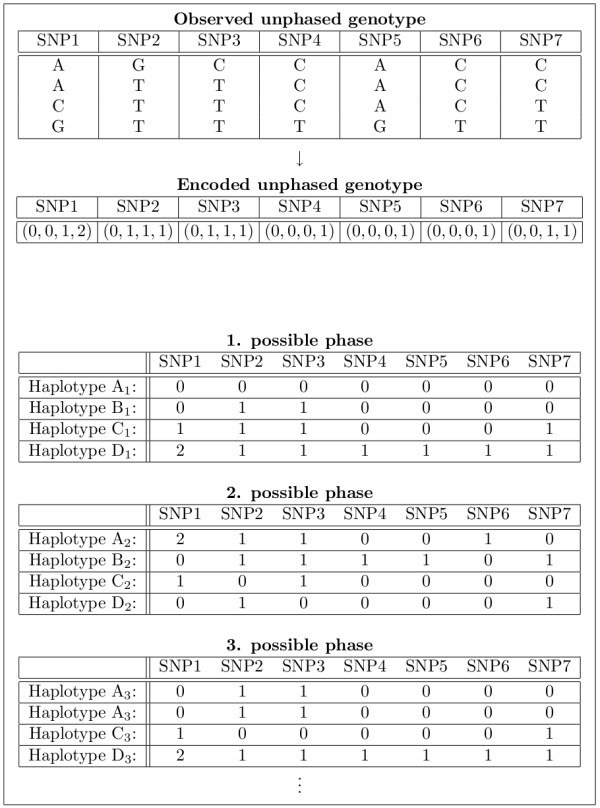
**Explanation of unphased genotypes**. The example shows an unphased tetraploid genotype at the top, which consists of three different alleles at the first SNP site and two different alleles at the remaining sites. In the second table, the given genotype is given in its vector representation. Different nucleotides at different SNP sites may be encoded by identical numbers, which depends on the allele composition of the SNP sites. There is an exponential number of possible haplotype explanations for one unphased genotype. Below, three of the possible haplotype explanations are shown. The first explanation can be resolved directly from the vector representation of the unphased genotype. The second explanation is resolved by randomly permutating the allele arrangement of the SNP sites. Finally, the third explanation is outstanding since it uses only three different haplotypes. Note that at least the first SNP site of the example will be further encoded as described in "Extension to SAT model for polyallelic polyploids".

A tetraallelic SNP site of a tetraploid individual at which two alleles occur once and a third occurs twice is encoded as a vector (0, 1, 2, 2), (0, 1, 3, 3), (0, 2, 3, 3) or (1, 2, 3, 3) (in fact there are twelve possible encodings, see Table 1). Finally, there is only one possible encoding for a tetraallelic SNP site of a tetraploid individual at which all four alleles occur: (0, 1, 2, 3). Triallelic and biallelic SNP sites are encoded accordingly as presented in Table 1. Then, explanation of a genotype is defined as: if *p *haplotypes explain an unphased genotype *g*_*i*_, the *p *haplotypes and the unphased genotype *g*_*i *_show the same allele composition at each SNP site (Figure 2).

One of the approaches to the haplotype inference problem is called Haplotype Inference by Pure Parsimony [5]. A solution to this problem minimises the total number of distinct haplotypes used. The SAT-based formulation of the HIPP models whether there is a set H of *r *distinct haplotypes, with *r *= |H| haplotypes, such that each genotype *g*_*i *_∈ G is explained by *p *haplotypes in H. The SAT-based algorithm considers increasing sizes for H, from a lower bound *lb *to an upper bound *ub *[7]. Trivial lower and upper bounds are, respectively, 1 and *pn*. The algorithm terminates for a size of H for which there are *r *= |H| haplotypes such that every genotype in G is explained by *p *haplotypes in H. The smallest *r *for which such a set H exists is a most parsimonious set of explaining haplotypes.

All variables of the Boolean satisfiability problem are two-valued. Depending on the truth assignment, a Boolean formula is either *true *or *false*. Then, SAT consists of the determination if an assignment to a given Boolean formula in conjunctive normal form (CNF) exists such that the formula evaluates to *true*, or the proof that such an assignment does not exist. Solving SAT is NP-complete [6]. The Boolean satisfiability problem for HIPP, however, can efficiently be solved [7] by SAT solvers such as MiniSat [11,12], MiraXT [13] or Sat4J [14]. This may be explained by a unknown hidden structure in the genotype data, which makes the problem easier to solve.

### SAT model for biallelic polyploids

The first SAT formulation for HIPP was introduced in [7] and the presented constraints were implemented in the software SHIPs [15]. Unfortunately, this approach is restricted to diploid and biallelic species. Here, we extend the formulation of constraints from [7] to polyploid biallelic populations of genotypes. In a tetraploid, biallelic population of genotypes, the possible alleles are modeled by 0 or 1 respectively (e.g. SNP site *j *of individual *i *with *g*_*i*, *j *_= (0, 1, 1, 1), $g_{i,j}^{1}$ = 0, $g_{i,j}^{2}$ = 1, $g_{i,j}^{3}$ = 1 and $g_{i,j}^{4}$ = 1). Furthermore, the haplotypes can be modeled such that *h*_*k*, *j *_∈ {0, 1}, where *h*_*k*, *j *_denotes the *j*^*th *^site of haplotype *k*. A haplotype *h*_*k *_can then be viewed as a binary word *h*_*k*,1 _... *h*_*k*, *m *_of length *m *over the alphabet {0, 1}.

For a given value of *r*, the model considers *r *haplotypes and aims at finding *p *haplotypes (which can possibly represent the same haplotype) with each genotype *g*_*i*_. As a result, for each genotype *g*_*i*_, the model uses selector variables for selecting which haplotypes are used for explaining *g*_*i*_. Since the genotype is to be explained by *p *haplotypes, the model uses *p *sets of *r *selector variables, $s_{k,i}^{l}$. Hence, genotype *g*_*i *_is explained by haplotypes $h_{k_{1}},...,h_{k_{p}}$,if $s_{k_{1},i}^{1}=1,...,s_{k_{p},i}^{p}=1$.

If the sum of the elements of binary vector *g*_*i*, *j *_equals 0, then $g_{i,j}^{l}$ = 0, with 1 ≤ *l *≤ *p*. Then the model requires that the following is satisfied:

(1) $$(\neg h_{k,j}\vee \neg s_{k,i}^{l}),$$

where 1 ≤ *k *≤ *r *and 1 ≤ *l *≤ *p*. Hence, if haplotype *k *is selected for explaining genotype *i*, by at least one of the *p *representatives, then the value of haplotype *k *at site *j must *be 0. If the sum of the elements of binary vector *g*_*i*, *j *_equals *p*, then $g_{i,j}^{l}$ = 1, with 1 ≤ *l *≤ *p*. Then the model requires that the following is satisfied:

(2) $$(h_{k,j}\vee \neg s_{k,i}^{l}),$$

where 1 ≤ *k *≤ *r *and 1 ≤ *l *≤ *p*. Hence, if haplotype *k *is selected for explaining genotype *i*, by at least one of the *p *representatives, then the value of haplotype *k *at site *j must *be 1.

Otherwise, if the sum of the elements of binary vector *g*_*i*, *j *_does not equal 0 nor *p*, one requires that the haplotypes explaining genotype *g*_*i *_show the corresponding number of 1s and 0s at site *j*. This is achieved by creating *p *variables $g_{i,j}^{1},...,g_{i,j}^{p}$ where $g_{i,j}^{l}$ ∈ {0, 1}, which represent the possible arrangements of 1s and 0s at site *j*. In the diploid situation, the model requires two clauses in CNF:

(3) $$(g_{i,j}^{1}\vee g_{i,j}^{2})\wedge (\neg g_{i,j}^{1}\vee \neg g_{i,j}^{2}).$$

Formula 3 evaluates to *true *iff one of the possible allele arrangements of a heterozygous and diploid SNP site is assigned to the Boolean variables. Thus, the formula is equivalent to the enumeration of all possible allele arrangements at the given SNP site, where an arrangement is given as conjunction of literals and all arrangements are connected by disjunctions (disjunctive normal form):

(4) $$(\neg g_{i,j}^{1}\wedge g_{i,j}^{2})\vee (g_{i,j}^{1}\wedge \neg g_{i,j}^{2}).$$

For *p *> 2 it is also straightforward to formulate the corresponding constraints in a disjunctive normal form (DNF) by enumerating all allele arrangements analogously to Formula 4. Each formula in DNF can be transformed into an equivalent formula in CNF [16]. As a result, for the case where *g*_*i*, *j *_is a heterozygous SNP site, then the model requires that the following is satisfied:

(5) $$(h_{k,j}\vee \neg g_{i,j}^{l}\vee \neg s_{k,i}^{l})\wedge (\neg h_{k,j}\vee g_{i,j}^{l}\vee \neg s_{k,i}^{l}),$$

where 1 ≤ *k *≤ *r *and 1 ≤ *l *≤ *p*. For each *i *and *l*, it is necessary that exactly one haplotype is used, and so exactly one selector variable be assigned value 1. For 1 ≤ *l *≤ *p*, this can be captured with cardinality constraints:

(6) $$\left(\sum_{k=1}^{r}s_{k,i}^{l}=1\right).$$

**Figure 3:**
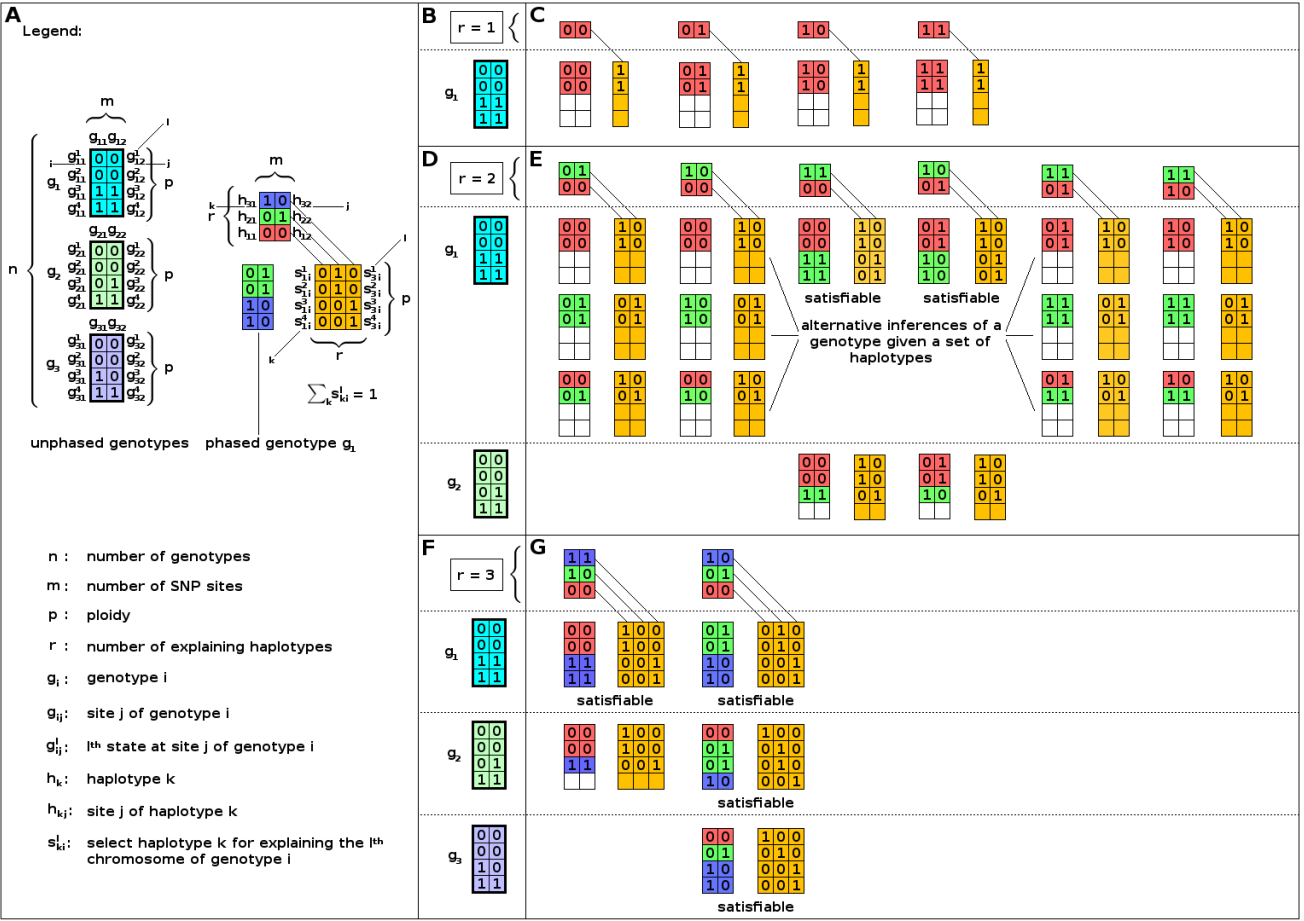
**Usage of Boolean variables**. Panel A shows a population of three unphased genotypes. Each of the tetraploid biallelic genotypes consists of two SNP sites. Variable names and usage of indices are given. Colour of genotype represents the coloured mixture of the explaining haplotypes. Yellow variables represent selection variables. In the model, phased genotypes are represented by selection variables. However, the possible phase of genotypes (explanation by haplotypes) is also shown. Panel B to G are read from left to right and top to bottom. If a genotype can be explained by four haplotypes from a given a set of haplotypes, it is tried to explain the next genotype in the population. Panel B and C: one haplotype is assumed as most parsimonious set and it is tried to explain genotype *g*_1_. Above the dashed line, the assumed *r *haplotypes and all four possible most parsimonious sets are shown. Below the dashed line, unphased genotype *g*_1 _and each try to explain genotype *g*_1 _by the assumed set of haplotypes is given. If no satisfying assignment can be found, corresponding cells are not filled with Boolean values. Relationships between explaining haplotypes and the corresponding column of selection variables is symbolised by diagonal lines. Rows of selection variables are constrained to sum up to 1 since exactly one haplotype is to be selected for representing a chromosome. Panel D and E: two haplotypes are assumed as most parsimonious set. All possible sets of haplotypes are shown. Strict lexicographic order breaks symmetries in sets of explaining haplotypes. The third and fourth set of explaining haplotypes can explain genotype *g*_1 _but fail to explain genotype *g*_2_. Panel F and G: three haplotypes are assumed as most parsimonious set. The first set is not able to explain the whole population. In contrast, the second set is able to explain all three genotypes. Thus, this set solves the HIPP for the given population.

These sums can be formulated in CNF by the utilization of an additional variables $d_{k,i}^{l}$ which corresponds to the Boolean state if a haplotype was already selected. The model requires that the following is satisfied:

(7) $$\begin{array}{ll} d_{1,i}^{l}\Leftrightarrow s_{1,i}^{l} & \\ \neg d_{k,i}^{l}\vee \neg s_{k+1,i}^{l} & 2\leq k\leq r-1\\ d_{k+1,i}^{l}\Leftrightarrow (d_{k,i}^{l}\vee s_{k+1,i}^{l}) & 1\leq k\leq r-1\\ d_{r,i}^{l}=1, & \end{array}$$

where 1 ≤ *l *≤ *p*. Figure 3 illustrates how the variables are used for explaining unphased genotypes.

### Efficient method for obtaining the model for biallelic polyploids

For the case *p *> 2, it is straightforward to formulate the constraints for the $g_{i,j}^{l}$ variables from heterozygous SNP sites in DNF by enumerating all allele arrangements. Each formula in DNF can be transformed into an equivalent formula in CNF using Tseitin's transformation [16]. However, the enumeration of all arrangements is of exponential complexity. Our objective here is to find an equivalent representation of enumeration of arrangements. This representation is to be in CNF and to allow formulation in polynomial time. Combinatorial problems as described above can also be represented by sums. For instance, for an individual from a tetraploid species with two 0 and two 1 alleles at a biallelic SNP site, all six allele arrangements are determined if the sum of the elements of a binary vector that represents the allele composition is constrained to 2: (0, 0, 1, 1), (0, 1, 0, 1), (1, 0, 0, 1), (1, 0, 1, 0), (1, 1, 0, 0) and (0, 1, 1, 0).

In the following, we combine simple logical circuits, such as *half adders *and *full adders *to derive general summation constraints which can be formulated in polynomial time. The usage of one full adder allows the summation of two bits where a full adder consists of two half adders. Formula 8 gives constraints for a half adder, where *A *and *B *are two bits which have to be summed, *C *is the resulting carry over and *S*_*half *_the resulting sum. Based on a half adder the constraints for a full adder can be derived as given in Formula 9. Variable *S*_*full *_is the complete sum of the two bits *A *and *B*, where the variable *C*^2 ^is the resulting carry over. Variable *C*^1 ^represents an additional bit that is added to the sum of *A *and *B*. Thus, the total sum of *A*, *B *and *C*^1 ^is at least 0 and at most 3. The first bit of the result is stored in *S*_*full *_and the second bit in *C*^2^.

(8) $$\begin{array}{c} (A\wedge B)\Leftrightarrow C\\ \neg \left(\left(A\wedge B)\vee \neg (A\vee B\right)\right)\Leftrightarrow S_{half} \end{array}$$

(9) $$\begin{array}{c} \left(\left(A\wedge B)\vee (S_{half}\wedge C^{1}\right)\right)\Leftrightarrow C^{2}\\ \left(\left(\neg S_{half}\vee \neg C^{1})\wedge (S_{half}\vee C^{1}\right)\right)\Leftrightarrow S_{full} \end{array}$$

**Figure 4:**
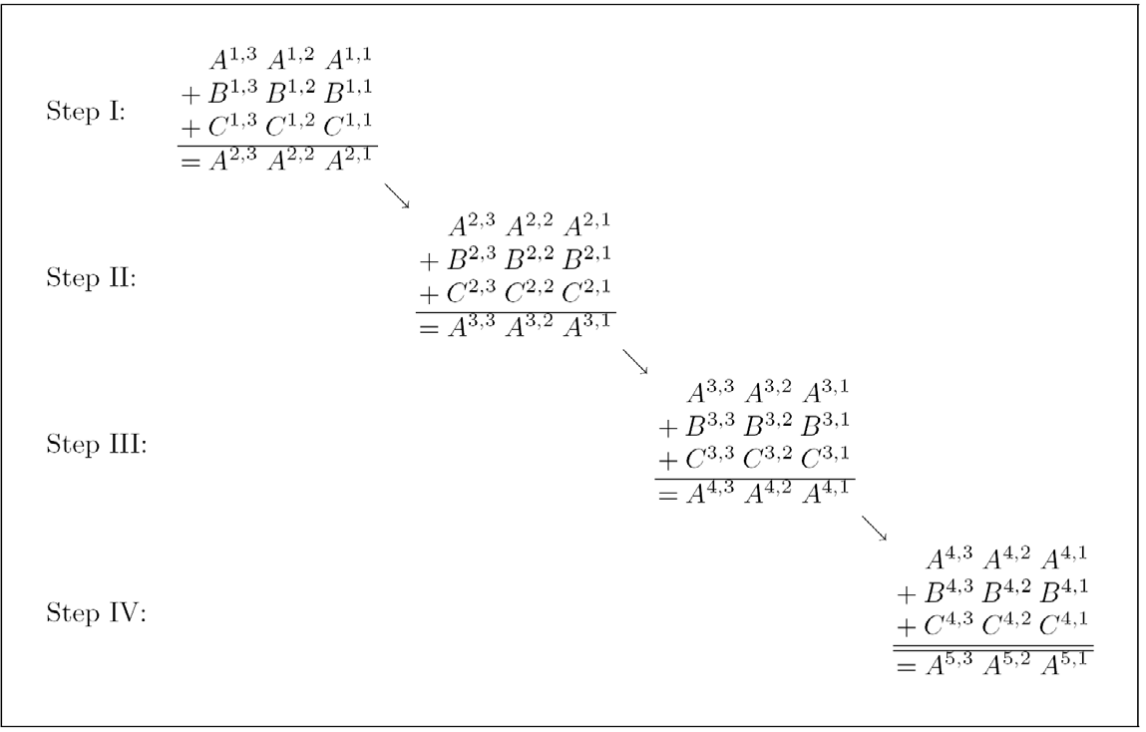
**Example of naive summation based on a ripple carry adder**. The *A *variables are used for storing the accumulation of the sum. The *A*^1, *t *^variables, with 1 ≤ *t ≤ *3, are constrained to zero. Additionally, the *C*^*l*,1 ^and the *B *variables, except the *B*^*l*,1 ^variables, with 1 ≤ *l *≤ 4, are also constrained to zero. The *S *variables are set to the binary representation of the required sum, e.g. if *S *= (1, 0, 0), the only satisfying assignment of Boolean values to the *B*^*l*,1 ^variables is (1, 1, 1, 1). If *S *= (0, 1, 0), there are six satisfying assignments to the *B*^*l*,1 ^variables, namely (1, 1, 0, 0), (1, 0, 1, 0), (1, 0, 0, 1), (0, 1, 0, 1), (0, 0, 1, 1) and (0, 1, 1, 0).

The *C*^2 ^carry over of a first full adder may be connected with the *C*^1 ^carry over of a second full adder. Analogously, the *C*^2 ^carry over of the second full adder may be connected with a third full adder and so on. If *w *full adders are connected in this way, the result represents a *ripple carry adder *that is able to sum up two *w *bit words. To describe the necessary *w *carry overs, the model requires that the following is satisfied:

(10) $$\left(\left(A^{t}\wedge B^{t})\vee (S_{half}^{t}\wedge C^{t}\right)\right)\Leftrightarrow C^{t+1},$$

where *t *= 1, ..., *w *- 1. Analogously, to describe the necessary *w *full adders, the model requires that the following is satisfied:

(11) $$\left(\left(\neg S_{half}^{t}\vee \neg C^{t})\wedge (S_{half}^{t}\vee C^{t}\right)\right)\Leftrightarrow S_{full}^{t},$$

where *t *= 1, ..., *w*. The number of necessary bits is at most *w *= ⌊log_2 _*p*⌋ + 1 if the binary vector which is summed up has length *p*. Thus, *p *+ 1 different *A *vectors and *p *different *B*, *C *and *S*_*full *_vectors are needed. Furthermore, the $S_{full}^{l,t}$ variables are replaced by the *A*^*l*+1, *t *^variables such that only the *A*^*p*+1, *t *^vector is left for constraining the sum, where 1 ≤ *l *≤ *p *and 1 ≤ *t *≤ *w*. It follows that summing can be represented as shown in Figure 4.

A simplification is achieved if vector *C*^*l*,1^, where 1 ≤ *l *≤ *p*, (Figure 4) contains the possible allele arrangements [17] and the *A *vectors store the accumulation of the sum. In this situation, all *B *variables can be set to zero. The constraints of carry overs reduce to:

(12) (*A*^*l*, *t *^∧ *C*^*l*, *t*^) ⇔ *C*^*l*, *t*+1^,

where 1 ≤ *l *≤ *p*. Additionally, if $S_{full}^{l,t}$ variables are replaced by *A*^*l*+1, *t *^variables, the sums reduce to:

(13) ((¬*A*^*l*, *t *^∨ ¬*C*^*l*, *t*^) ∧ (*A*^*l*, *t *^∧ *C*^*l*, *t*^)) ⇔ *A*^*l*+1, *t*^,

where 1 ≤ *l *≤ *p*. Reformulating the constraints from Formula 12 into CNF yields the following for the carry overs:

(14) $$\begin{array}{ccc} (\begin{array}{cc} \neg A^{l,t} & \end{array} & \begin{array}{cc} \vee & \neg C^{l,t}\vee C^{l,t+1} \end{array} & )\wedge \\ (\neg C^{l,t+1} & \begin{array}{cc} \vee & A^{l,t} \end{array} & )\wedge \\ (\neg C^{l,t+1} & \begin{array}{cc} \vee & C^{l,t} \end{array} & ). \end{array}$$

By reformulating the constraints from Formula 13 into CNF, we obtain the following expression for the sums:

(15) $$\begin{array}{cccccc} (\neg A^{l,t} & \vee & \neg C^{l,t} & \vee & \neg A^{l+1,t} & )\wedge \\ (\neg A^{l,t} & \vee & C^{l,t} & \vee & A^{l+1,t} & )\wedge \\ (A^{l,t} & \vee & \neg C^{l,t} & \vee & A^{l+1,t} & )\wedge \\ (A^{l,t} & \vee & C^{l,t} & \vee & \neg A^{l+1,t} & ). \end{array}$$

For each individual and SNP site in a biallelic population, variables corresponding to *A *and *C *need to be defined. Let variables $a_{i,j}^{l,t}$, with 1 ≤ *l *≤ *p *+ 1 and 1 ≤ *t *≤ *w*, denote the accumulation of the sum.

Additionally, let variables $c_{i,j}^{l,t}$, with 1 ≤ *l *≤ *p *and 1 ≤ *t *≤ *w*, stand for the carry overs. For a SNP site *j *from genotype *i*, summing constraints can easily be obtained by replacing the $c_{i,j}^{l,1}$ variables with the $g_{i,j}^{l}$ variables [17], with 1 ≤ *l *≤ *p*. Finally, the $a_{i,j}^{p+1,t}$ variables, with 1 ≤ *t *≤ *w*, are constrained to the binary representation of the required sum.

### Extension to SAT model for polyallelic polyploids

In this and the following sections we use notation ${\left(x\right)}_{2}^{y}$ to mean the *y*^*th *^bit of the binary encoded number *x*. Furthermore, we use a function *b*(*A, B*) to substitute variables:

(16) $$b(A,B)=\{\begin{array}{ll} A & \text{if }B=1\\ \neg A, & \text{otherwise}. \end{array}$$

Dependent on the input, Function 16 defines if the substituted variable is negated.

The $g_{i,j}^{l}$ variables are insufficient for describing arrangements of more than two alleles at a SNP site. The representation of, for instance, three different states needs at least two bits. We define *o*_*j *_as the number of different alleles from all individuals at SNP site *j *of a population of genotypes. If *o*_*j *_> 2, the representation of the SNP site is extended to *w*_*j *_= ⌈log_2 _*o*_*j*_⌉ binary columns. Thus, $g_{i,j}^{l}$ is split to $g_{i,j}^{l,1},...,g_{i,j}^{l,w_{j}}$. Each allele is encoded by its corresponding binary number. For instance, the four alleles 0, 1, 2 and 3 at a tetraallelic SNP site are encoded as 00, 01, 10 and 11, respectively. The haplotypes are then extended analogously, such that $h_{k,j}\in {\left\{0,1\right\}}^{w_{j}}$ denotes the *j*^*th *^site of haplotype *k*.

For generalisation to polyallelic SNP sites, the formulation of binary sums can be reused. Let $z_{i,j}^{u_{j}}$ be the number of allele *u*_*j *_at a specific SNP site *j *in unphased genotype *i*, where 1 ≤ *u*_*j *_≤ *o*_*j*_. The value of *o*_*j *_can be greater than *p *but $\sum_{u_{j}=1}^{o_{j}}z_{i,j}^{u_{j}}=p$. For a set of nucleotide sequences, it holds that *o*_*j *_≤ 4.

For each individual and SNP site, *o*_*j *_vectors *v*_*i*, *j *_of length *p *are defined. Variable $v_{i,j}^{l,u_{j}}$ is set to 1 if allele *u*_*j *_is represented at position *l *by its corresponding binary number:

(17) (b(gi,jl,1,(uj−1)12)∧b(gi,jl,2,(uj−1)22)∧⋮b(gi,jl,wj,(uj−1)2wj))⇔vi,jl,uj,

where 1 ≤ *l ≤ p *and 1 ≤ *u*_*j *_≤ *o*_*j*_. It is necessary to formulate the sums $\sum_{l=1}^{p}v_{i,j}^{l,u_{j}}$ which must equal $z_{i,j}^{u_{j}}$, as described in the previous sections such that each allele at SNP site *j *occurs $z_{i,j}^{u_{j}}$ times in genotype *i*.

**Figure 5:**
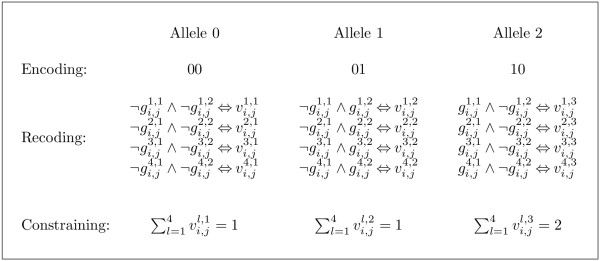
**Example showing constraints of triallelic SNP site**. Assume a triallelic SNP site and an individual with the allele composition (0, 1, 2, 2). First, the alleles are encoded to their corresponding binary representation, here 0 ≡ 00, 1 ≡ 01 and 2 ≡ 10. Second, each allele is recoded to binary variables $v_{i,j}^{l,1}$, $v_{i,j}^{l,2}$ and $v_{i,j}^{l,3}$, with 1 ≤ *l *≤ 4. Finally, the three vectors are constrained to the corresponding sum.

An example is shown in Figure 5. Generally, at a given SNP site of an individual, there can be at most *p *different alleles, even if the number of alleles in the population at this site is *o*_*j *_> *p*. Thus, only the constraints for *p *sums have to be given.

In variables $v_{i,j}^{l,u_{j}}$, with 1 ≤ *l *≤ *p*, the arrangements of allele *u*_*j *_are represented, where allele *u*_*j *_is encoded as 1 and all other alleles are encoded as 0s. In contrast, the $g_{i,j}^{l,t_{j}}$ variables, with 1 ≤ *l *≤ *p *and 1 ≤ *t*_*j *_≤ *w*_*j*_, represent the arrangements of all alleles at the given SNP site. It is still necessary that the correct haplotypes $h_{k_{1}},...,h_{k_{p}}$ are chosen to explain the alleles at *g*_*i*, *j*_. Then the model requires that the following is satisfied:

(18) $$\begin{array}{llllll} (h_{k,j}^{t_{j}} & \vee & \neg g_{i,j}^{l,t_{j}} & \vee & \neg s_{k,i}^{l} & )\wedge \\ (\neg h_{k,j}^{t_{j}} & \vee & g_{i,j}^{l,t_{j}} & \vee & \neg s_{k,i}^{l} & ), \end{array}$$

where 1 ≤ *t*_*j *_≤ *w*_*j*_, 1 ≤ *k *≤ *r *and 1 ≤ *l *≤ *p*.

The final (most parsimonious) number of haplotypes is denoted by *r*_*f *_and the maximal number of alleles, which is found at a SNP site of the considered population, is denoted by *o*_*max*_. Then, if *p *log_2 _*p *≤ *r*_*f *_log_2 _*o*_*max*_, the number of variables (Table 2) and constraints in the proposed model is, respectively, O(*n m p*^2 ^log_2 _*p*) and O(*r*_*f *_*n m p *log_2 _*o*_*max*_) . The complexity for the variables and constraints decreases to O(*n m p *log_2 _*p*) and O(*r*_*f *_*n m p*) if the nucleotide alphabet is considered.

**Table 2:** Complexity of the model

	Number of variables
*h*	*r*_*f *_*m *log_2 _*o*_*max*_
*g*	*n m p *log_2 _*o*_*max*_
*s*	*r*_*f *_*n p*
*a*	*n m p*^2 ^log_2 _*p*
*c*	*n m p*^2^log_2 _*p*
*v*	*n m p*^2^

The complexity of the presented model is listed below. Variable *r*_*f *_is the final value of *r*. The maximal number of alleles, which is found at a SNP site of the considered population, is denoted by *o*_*max*_. The number of variables is O(*n m p*^2 ^log_2 _*p*). If *p *log_2 _*p *≤ *r*_*f *_log_2 _*o*_*max*_, the number of constraints is O(*r_f_ n m p *log_2 _*o*_*max*_).

### Constraints for breaking symmetries in haplotypes

It is important to note that the model proposed above is not practical for most existing problem instances, even with the most efficient SAT solvers [7]. This problem, however, can be solved by breaking symmetries to prune the search space. As described in [7,8], symmetries in explaining haplotypes can be broken by sorting the haplotypes lexicographically. A strict lexicographic ordering can be achieved by the formulation of constraints that become true if *h*_1 _is strictly smaller than *h*_2_, *h*_2 _is strictly smaller than *h*_3_, and so on. If the ordering is not strict it is not guaranteed that all explaining haplotypes are pairwise distinct.

For the ordering, *h*_*k*, *j *_is compared with *h*_*k*+1, *j*_, where 1 ≤ *k *≤ *r *- 1 and 1 ≤ *j *≤ *m*. Additionally, new variables *e*_*k*, *j *_have to be introduced, which record the value of *h*_*k*, *j *_<*h*_*k*+1, *j*_. Then, the model requires that the following is satisfied:

(19) $$\begin{array}{c} \begin{array}{ccc} \left(\neg h_{k,m}\wedge h_{k+1,m}\right) & \Leftrightarrow & e_{k,m}\wedge \\ \left((\neg h_{k,j}\wedge h_{k+1,j})\vee e_{k,j+1}\right) & \Leftrightarrow & e_{k,j}\wedge \end{array}\\ e_{k,1}=1, \end{array}$$

where 1 ≤ *j *≤ *m + *1. If an *e*_*k*, *j *_becomes true because the constraint *h*_*k*, *j *_<*h*_*k*+1, *j *_is satisfied, it is not necessary to compare *h*_*k*, *j*' _to *h*_*k*+1, *j*'_, where *j' *<*j*. We must, however, ensure that *h*_*k*, *j' *_≤ *h*_*k*+1, *j'*_, where *j' *> *j*. Then, the model requires that the following is satisfied:

(20) (¬*h*_*k*, *j *_∨ *h*_*k*+1, *j *_∨ *e*_*k*, *j*_),

where 1 ≤ *j *≤ *m*. If an assignment can be found such that all clauses in Formulas 19 – 20 are true, where 1 ≤ *k *≤ *r *- 1, the haplotypes are in lexicographical order.

### Constraints for breaking symmetries in genotypes

Haplotypes that infer a genotype can be lexicographically ordered in a way similar to the set of *r *explaining haplotypes [7,8]. The *l*^*th *^haplotype inferring an unphased genotype *i *is marked by a binary variable $s_{k,i}^{l}$. The sum $\sum_{k=0}^{r}s_{k,i}^{l}$ is constrained to equal 1 so that exactly one haplotype is selected for explaining the *l*^*th *^row of *g*_*i*_. In contrast to the most parsimonious set of explaining haplotypes, the selection variables have to be ordered non-strict lexicographically since homozygous genotypes can not be explained by sets of pairwise distinct haplotypes.

As a result, the *p *selection vectors of each genotype *g*_*i *_can be ordered lexicographically by constraining the vectors by $s_{i}^{1}\leq s_{i}^{2}\leq \ldots \leq s_{i}^{p-1}\leq s_{i}^{p}$[7,8]. For this purpose, the constraints of ordering the haplotypes are reused and slightly changed. New variables $f_{k,i}^{l}$ have to be introduced, which record the value of $s_{k,i}^{l}<s_{k,i}^{l+1}$. Then, the model requires that the following is satisfied:

(21) $$\begin{array}{lll} \left(\neg s_{r,i}^{l}\wedge s_{r,i}^{l+1}\right) & \Leftrightarrow & f_{r,i}^{l}\wedge \\ \left((\neg s_{k,i}^{l}\wedge s_{k,i}^{l+1})\vee f_{k+1,i}^{l}\right) & \Leftrightarrow & f_{k,i}^{l}, \end{array}$$

where 1 ≤ *k *≤ *r *+ 1. If an $f_{k,i}^{l}$ becomes true because the constraint $s_{k,i}^{l}<s_{k,i}^{l+1}$ is satisfied, it is not necessary to compare $s_{k^{\prime },i}^{l}$ to $s_{k^{\prime },i}^{l+1}$, where *k*' <*k*. We must, however, ensure that $s_{k^{\prime },i}^{l}\leq s_{k^{\prime },i}^{l+1}$, where *k*' > *k*. Then, the model requires that the following is satisfied:

(22) $$(\neg s_{k,i}^{l}\vee s_{k,i}^{l+1}\vee f_{k,i}^{l}),$$

where 1 ≤ *k *≤ *r*. Because it is almost the formulation of a strict lexicographic order, except that the variable $f_{1,i}^{l}$ does not have to be true, it has to be relaxed to become a non-strict order. This can be done by formulating the constraints for either vector $s_{i}^{l}$ is strict smaller than vector $s_{i}^{l+1}$ or both vectors are equal.

Finally, the model requires that the following is satisfied:

(23) $$(s_{k,i}^{l}\Leftrightarrow s_{k,i}^{l+1})\vee f_{1,i}^{l},$$

where 1 ≤ *k *≤ *r*. If an assignment can be found for which all clauses in Formulas 21 – 23 are true, where 1 ≤ *l *≤ *p *- 1, the selection variables of genotype *g*_*i *_are in non-strict lexicographic order.

### Constraints for alternative most parsimonious sets of haplotypes

There can be several most parsimonious sets of haplotypes which differ slightly and yet can explain the unphased genotype data. Call $H_{k,j}^{\text{i}}$ the set of previously found binary assignments to *h*_*k*, *j*_, where 1 ≤ i ≤ *N*_*alt *_and *N*_*alt *_is the number of previously found inferences. Constraints for alternative most parsimonious sets of haplotypes can be easily formulated by the exclusion of previously found sets. Then, the model requires that the following is satisfied:

(24) $$\begin{array}{c} \neg (b(h_{k,1},H_{k,1}^{\text{i}})\wedge \\ b(h_{k,2},H_{k,2}^{\text{i}})\wedge \\ \vdots \\ b(h_{k,m},H_{k,m}^{\text{i}})), \end{array}$$

where 1 ≤ *k *≤ *r *and 1 ≤ i ≤ *N*_*alt*_. Application of De Morgan's laws to Formula 24 results in Formula 25:

(25) $$\begin{array}{c} (\neg b(h_{k,1},H_{k,1}^{\text{i}})\vee \\ \neg b(h_{k,2},H_{k,2}^{\text{i}})\vee \\ \vdots \\ \neg b(h_{k,m},H_{k,m}^{\text{i}})), \end{array}$$

A nice feature of constraining alternative haplotype inferences is that Formula 25 is automatically in CNF.

### Constraints for alternative genotype inferences

Given *p *explaining haplotypes, there can also be alternative inferences of genotypes. Constraints of alternative genotype inferences are given similarly to the constraints of alternative haplotype inferences. Call $S_{k,i}^{l,\text{j}}$ the set of previously found binary assignments to the selection variables $s_{k,i}^{l}$ of genotype *i*, where 1 ≤ j ≤ *M*_*alt *_and *M*_*alt *_is the number of previously found inferences. Now consider only one previously found assignment to the selection variables of genotype *i *with $S_{k_{1,i},i}^{1}=1,...,S_{k_{p,i},i}^{p}=1$. Since it is sufficient to constrain selection variables to 0, which were set to 1 in a previously computed inference, the already found explanations of all unphased genotypes are excluded by:

(26) $$\begin{array}{ccc} \neg (s_{k_{1,1},1}^{1} & \wedge \ldots \wedge & s_{k_{p,1},1}^{p}\wedge \\ s_{k_{1,2},2}^{1} & \wedge \ldots \wedge & s_{k_{p,2},2}^{p}\wedge \\ & \vdots & \\ s_{k_{1,n},n}^{1} & \wedge \ldots \wedge & s_{k_{p,n},n}^{p}). \end{array}$$

Application of De Morgan's laws to Formula 26 results in Formula 27:

(27) $$\begin{array}{ccc} (\neg s_{k_{1,1},1}^{1} & \vee \ldots \vee & \neg s_{k_{p,1},1}^{p}\vee \\ \neg s_{k_{1,2},2}^{1} & \vee \ldots \vee & \neg s_{k_{p,2},2}^{p}\vee \\ & \vdots & \\ \neg s_{k_{1,n},n}^{1} & \vee \ldots \vee & \neg s_{k_{p,n},n}^{p}). \end{array}$$

Formula 27 is in CNF and no reformulation is necessary. Such clauses have to be given for each previously found genotype inference j.

For constraining alternative genotype inferences, it is very important that genotype symmetries are broken as shown in Section "Constraints for breaking symmetries in genotypes". If symmetries are not broken, and if an assignment to the $s_{k,i}^{l}$ variables is excluded for a given unphased genotype *g*_*i*_, the SAT solver can still report an assignment that represents a permutation of the excluded assignment. For instance, vector $s_{i}^{l_{1}}$ with length *r *is exchanged by vector $s_{i}^{l_{2}}$ with length *r*.

Note that the number of alternative genotype inferences is equal to or greater than the number of alternative most parsimonious sets of haplotypes, since each alternative set of haplotypes defines at least one inference of genotypes. As a result, we do not calculate complete alternative genotype inferences in this study. Instead, we introduce an optimisation method for genotypes, based on explaining haplotypes and bootstrapping (see Section "Bootstrapping" and Section "Optimisation of genotypes").

### Bootstrapping

All possible minimal inferences are treated equally by the SAT approach. It is unlikely that the first haplotype inference found is the most probable one under the assumed model and given data. It is also unlikely that the first haplotype inference is the inference with fewest differences compared to the real data. The question which haplotype inference should be taken for further analysis remains. There must be one or more inferences which are supported better by the input data. To introduce a quality measurement of the haplotypes and alternative inferences which have been calculated, a bootstrapping procedure is introduced as follows. Bootstrapping is widely used (e.g. in phylogenetic reconstruction [18]) for estimating properties of an estimator. Those properties are measured when sampling from an approximate distribution. One standard choice for an approximate distribution is the empirical distribution of the observed data. To use the bootstrap to assess the uncertainty of estimates of the phased genotypes, the data should be a series of independently sampled points. Here, we assume that haplotypes are drawn independently from a most parsimonious set of explaining haplotypes which is the base of the population of genotypes. Thus, the independently drawn haplotypes satisfy the independence assumptions of the bootstrap method.

**Figure 6:**
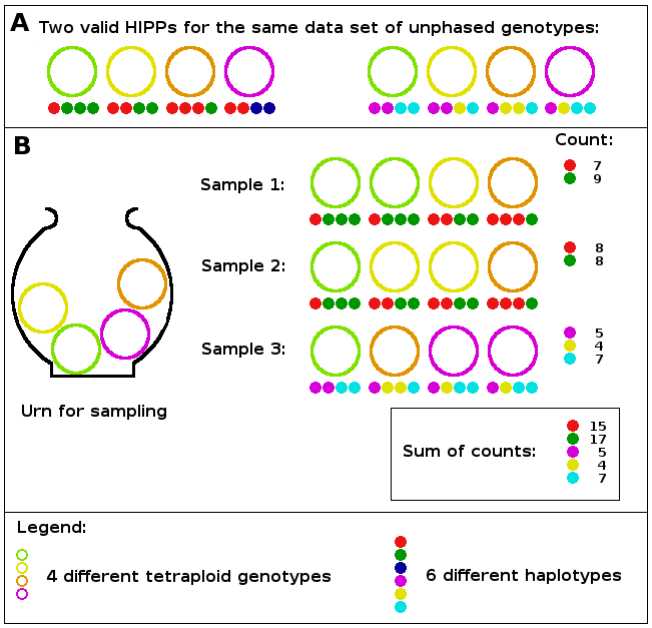
**Example of Bootstrapping**. Panel A shows two different most parsimonious inferences of four genotypes from a tetraploid species. different genotypes are represented by different coloured rings whereas different explaining haplotypes are represented by different coloured circles. In Panel B, the urn symbolises the population from which genotypes are sampled. Three bootstrap replicates are drawn with replacement, the genotypes are inferred, and the occurrences of the different haplotypes are counted (middle). Because of the parsimony criterion, the shown inference of sample 1 and sample 2 are unique. Sample 3 can alternatively be inferred with the green, red and blue haplotypes. The overall number of occurrences of the haplotypes is calculated. Finally, the two most parsimonious inferences are scored by the counts of haplotypes. The inference on the left has score 222 and the inference on the right has score 78. Inference on the left is selected for further analysis.

From a set G of *n *given unphased genotypes, a new set of *n *unphased genotypes $G^{\prime }$ is sampled by replacement (Figure 6). Sampled sets $G^{\prime }$ are inferred as described before, and each haplotype occurring in the inferences of the genotypes is added to a list. If a haplotype does not appear in the list, the haplotype and the number of its occurrences in the phased genotypes (its count) are added to the list. Otherwise, the former count of the haplotype in the list is increased by that number. For one $G^{\prime }$, this procedure is defined as one bootstrap replicate. Thus, the count of a haplotype, which is equivalent to a frequency, reflects its support by the input data and is defined as the haplotype's score. The process is repeated, and after a given number of bootstrap replicates, the corresponding counts of each haplotype occurring in the genotypes of an alternative haplotype inference are summed up. This sum is used to score the alternative haplotype inferences. The inference with the greatest score is assumed to be the one with the greatest support from the input data given the parsimony criterion.

### Optimisation of genotypes

**Figure 7:**
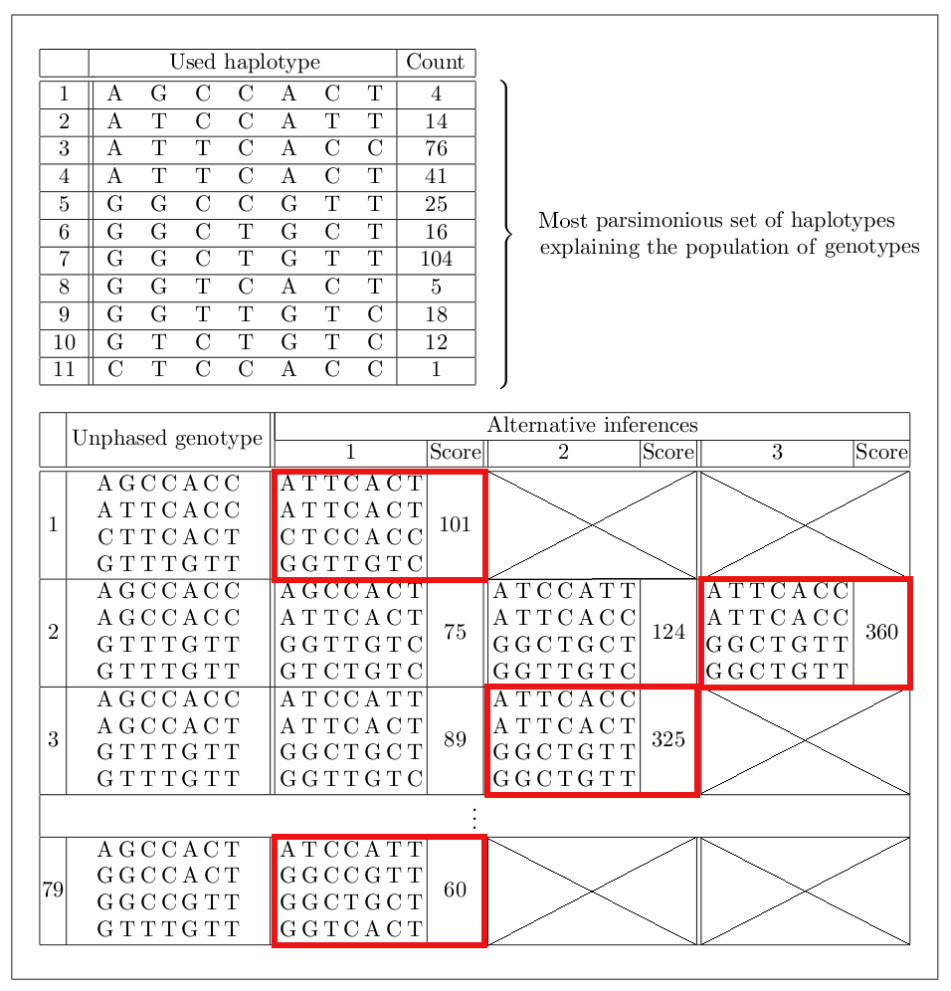
**Example of optimisation**. Based on computed alternative most parsimonious sets of haplotypes and bootstrapping scores, an optimisation of the phased genotypes is possible. For each most parsimonious set of haplotypes the algorithm therefore computes all alternative inferences of each unphased genotype. One most parsimonious set of explaining haplotypes and corresponding bootstrapping scores of some tetraploid data set are shown in the first table. The first column in the table below shows the unphased genotypes. The other columns contain alternative inferences and bootstrapping scores of the corresponding unphased genotype based on the explaining haplotypes. From the row of each genotype the highest scored phased genotype is selected (red). This forms a new inference of the original data with equal or better scoring.

Based on computed alternative most parsimonious sets of haplotypes and bootstrapping scores, an optimisation of the phased genotypes can be computed (Figure 7). For each most parsimonious set of haplotypes the algorithm therefore computes all alternative inferences of each unphased genotype, independent of the others. As mentioned above, this method only works if symmetries in genotypes are broken (if symmetries are not broken, the algorithm also reports genotype inferences that use same sets of haplotypes in different orders). This procedure results in a list of alternative phased genotypes for each original unphased genotype. Using the bootstrapping approach, each computed genotype is scored by the sum of the bootstrapping scores of its contained haplotypes. Alternatively, without bootstrapping, the frequencies of the haplotypes in the phased genotypes of all computed alternative inferences can be used similar to bootstrapping counts. Finally, from the alternative inference list of each genotype the highest scored genotype is selected in order to replace the former genotype in the inference. This forms a new inference of the original data with equal or better scoring.

The number of all alternative genotype inferences for a given most parsimonious set of haplotypes is the product of the alternative inferences of each genotype. Moreover, the product of the number of alternative genotype inferences from each alternative most parsimonious set of haplotypes is the number of all valid most parsimonious HIPPs.

### Calculation of lower and upper bounds

In contrast to integer linear programming formulations of HIPP [9,10], the SAT approach is not able to optimise a target function directly. Thus, each possible number of explaining haplotypes has to be tested incrementally starting with *r *= 1. Methods for the computation of lower and upper bounds [7,19] can be applied to avoid the iteration until a most parsimonious solution is found. Furthermore, a lower bound can be used for reducing the size of the model [7]. Genotypes which only can be explained by distinct sets of haplotypes are called *incompatible*. Incompatible genotypes can be used for deriving a lower bound such that the size of the model can be reduced by eliminating *s *variables and corresponding clauses.

In the existing version of SATlotyper, the computation of lower and upper bounds is not implemented. It was found empirically that, if the approach is able to find a most parsimonious set of haplotypes in reasonable time, it is also able to prove the unsatisfiability of smaller sets of haplotypes in reasonable time. Nevertheless, it is not clear how large the increase in solvable instances would be if a calculation of lower and upper bounds were used in haplotype inference of polyploids. The computation of upper and lower bounds according to [7,19] may be added to SATlotyper in future versions.

### Comparing inferences with real data

To define a standard for the measurement of an inference of haplotypes and corresponding genotypes, we sum up the differences between genotypes from inference and corresponding genotypes from real data. The number of differences is defined as the distance *d *between both sets.

Call *G*_*i *_= $h_{k_{1}},...,h_{k_{p}}$ the inference of an unphased genotype by the SAT approach and ${G^{\prime }}_{i}=\{{h^{\prime }}_{{k^{\prime }}_{1}},...,{h^{\prime }}_{{k^{\prime }}_{p}}\}$ the real data for instance from simulation. Every haplotype *h*_*k *_in the inference has a counterpart ${h^{\prime }}_{k^{\prime }}$ in the real data set. The distance is defined recursively as shown in Formula 28 and Formula 29, where *d*_*ham*_(*h*_*k*_, *h'*_*k'*_) is the Hamming distance between *h*_*k *_and *h'*_*k'*_.

(28) $$D(G,G^{\prime })=\{\begin{array}{ll} 0, & \begin{array}{c} \text{if }G=\{\}\\ \vee G^{\prime }=\{\} \end{array}\\ \begin{array}{l} h\in G\wedge \\ \forall h^{\prime }\in G^{\prime }:\\ d_{ham}(h,h^{\prime })+\\ D(G\backslash h,G^{\prime }\backslash h^{\prime }) \end{array} & \text{otherwise} \end{array}$$

(29) *d *= min (*D*(*G*, *G*'))

The complexity of calculating *d *is exponential but for small *p *this is still possible.

### Software realisation

SATlotyper is implemented in Java and realises the constraints described above. Additionally, there are some obvious improvements included in the program, such as converting the $v_{i,j}^{l,u_{j}}$ vectors, with 1 ≤ *l *≤ *p*, to the corresponding Boolean inverse if min(*q*, *p *- *q*) = *p *- *q*, where *q *is the number of 1s. Another improvement is the enumeration of constraints for a SNP site such that instead of *o*_*j *_only *o*_*j *_- 1 sums have to be given if *o*_*j *_≤ *p*.

The SAT approach that we generalised [7] can not optimise a target function directly (but there are efforts to combine ILP and SAT features [12,17,20,21]). Therefore, the SAT formulation of an assumed number of explaining haplotypes has to be tested for satisfiability by the SAT solver. If it fails, the number of explaining haplotypes is incremented and then tested again. This is repeated until the SAT solver reports satisfiability. For unphased genotypes, given in CSV format, the program generates corresponding constraints and writes the resulting formula in CNF format to the file system. Next, the binary of the corresponding SAT solver is executed with the newly generated CNF file as input. After successful termination of the solver, SATlotyper reads, analyses and reports the output of the solver in XML format (Additional file 1).

SATlotyper is able to execute different SAT solvers and was tested with MiniSat [11,12], MiraXT [13] (a multithreaded SAT solver) and Sat4J [14] but can be easily adapted to other solvers accepting standard CNF file format. Access to single Boolean variables is realised by a hash which contains corresponding matrices. This object allows indexing by means of the corresponding keyword, for instance "haplotype", for a given type of variable.

## Results

The following results were computed on a laptop with 2048 MB RAM and AMD Turion™ 64 X2 Mobile Technology TL-56 (2 × 1.80 GHz). The operation system was a Linux system (Debian 4.0 ("*etch*")), kernel version 2.6.18-5-amd64. MiniSat 2 (minisat2-070721.zip [11,12]) was used for solving SAT.

### Development of SATlotyper

The presented generalisation of the original SAT approach [7] led to the development of SATlotyper, which can infer polyploid and polyallelic input. The SATlotyper algorithm is able to handle incomplete data sets where SNP sites are partly missing, without bringing in unjustified assumptions. For instance, SNP sites are missing when genotypes are heterozygous for alleles with indels (insertions or deletions) that may result in an interruption of analysable sequence data. Unknown sites are marked "N". With the SAT approach, no assumptions are made for individuals that contain SNP sites with no information available, i.e. the formulation of constraints for the corresponding individual and SNP site is omitted. If the formulation of constraints for a site is omitted, the SAT solver uses a set of haplotypes inferred from other unphased genotypes, provided that these haplotypes are compatible with the known sites of the unphased genotype containing missing information. The choice of haplotypes for explaining such a genotype is independent of the alleles that the explaining haplotypes show at the site with missing information.

### Testing SATlotyper on simulated data

In order to test SATlotypers performance, we simulated haplotypes comprising six SNP sites for ten tetraploid, biallelic populations with 100 individuals each. For every population, six simulated haplotypes were used as a pool for further simulation. These six different haplotypes of one population were sampled uniformly to generate a population of tetraploid individuals. The alleles of these haplotypes were also sampled uniformly. The simulation resulted in ten data sets of 100 individuals each.

In order to simulate noise in the simulated data sets, data changes were introduced by conversion of a randomly chosen nucleotide of a randomly chosen individual at a randomly chosen SNP site to the other allele at the same site. All manipulated SNP sites were marked and no longer changed. If, however, a change has to be introduced and the random procedure selects an already manipulated individual and site, the noise-procedure is repeated until the needed change is introduced. Homozygous SNP sites were excluded from change on the assumption that these sites are correctly analysed in real data sets. Moreover, heterozygous SNP sites were not changed to homozygous sites. Thus, *x*% of noise means exactly *x*% of changed SNP sites in the data set. The ten simulated data sets were modified by noise, where noise was increased from 0% to 10% in steps of 1% resulting in 110 different data sets. Four types of analysis were performed with the simulated data sets (Table 3). These four methods are referred to as Method 1–4 and are computed as follows.

**Table 3:** Comparison of the different methods of SATlotyper

Method	Features of SATlotyper		
	
	Alt. expl. hap.	Bootstrapping	Optimisation
1	No	No	No
2	Yes	Yes	No
3	Yes	Yes	Yes
4	No	No	Yes

**Figure 8:**
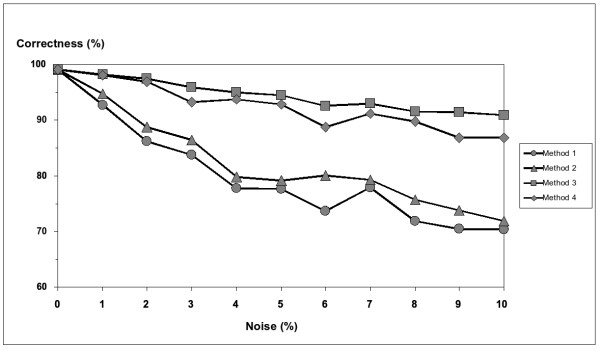
**Comparison of four types of analysis using simulated data sets**. For ten tetraploid, biallelic populations with 100 individuals each, haplotypes comprising six SNP sites were simulated. 0–10% noise was added to the data. Unphased simulated data sets were analysed with SATlotyper using four different methods: 1) first inference without optimisation; 2) alternative haplotype inferences scored by bootstrapping, selection of the best scored; 3) alternative haplotype inferences scored by bootstrapping, further optimisation of genotype inference; 4) first haplotype inference with optimisation of genotype inference. On the basis of the Hamming distance between predicted and original simulated genotype inferences, the correctness was calculated, normalised and plotted against the noise. Every data point represents the mean value of ten populations for the respective value of noise.

**Figure 9:**
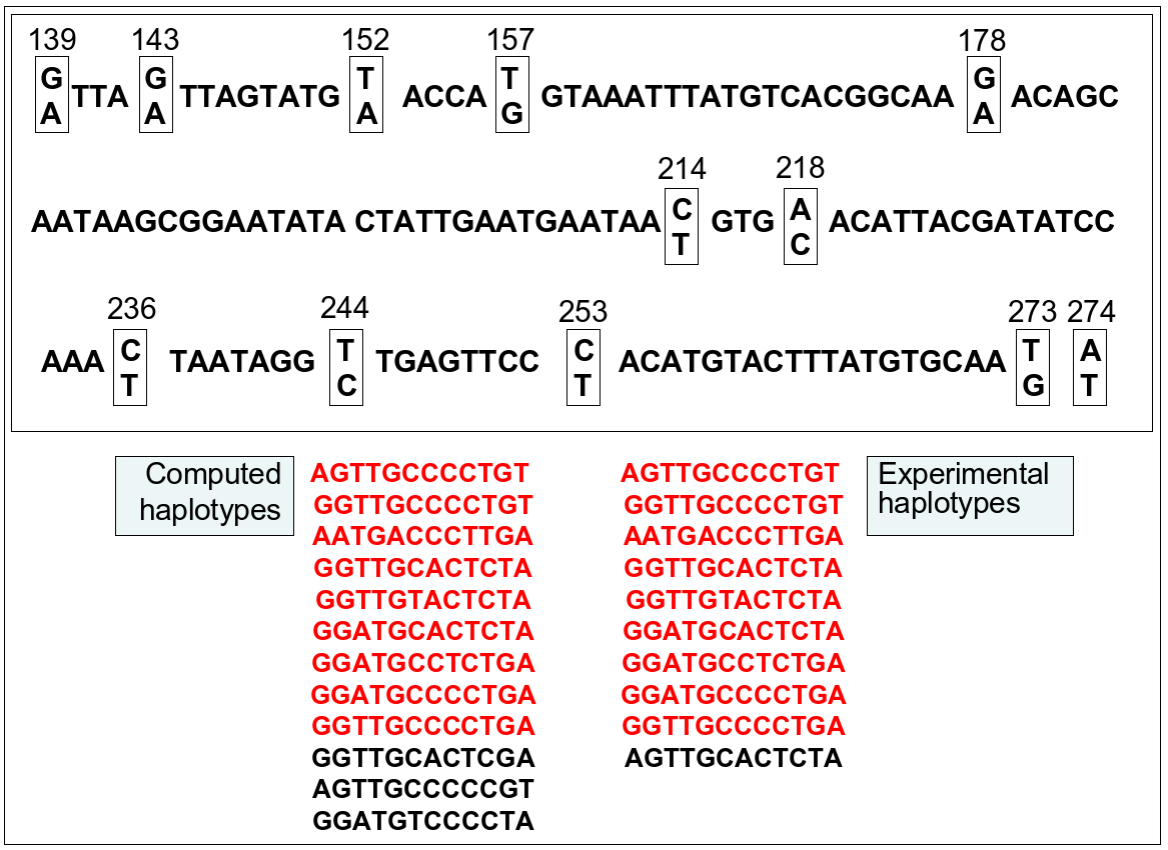
**Comparison of computational and experimental haplotypes for potato locus BA213c14t7**. Sequence of the potato locus *BA213c14t7*. The evaluated SNP sites are indicated by boxes. For a sub-population of nineteen individuals, haplotype sequences were identified computationally with Method 2 and experimentally by amplicon cloning and sequencing. The nine haplotypes identified by both methods are displayed in red.

The different features of SATlotyper are listed below. For the different results see also Figure 8, Figure 9 and Figure 12. Alt. expl. hap., computation of alternative most parsimonious sets of explaining haplotypes.

Method 1: for each data set exactly one haplotype inference was calculated.

Method 2: for each data set up to 250 alternative most parsimonious sets of explaining haplotypes and the corresponding haplotype inferences were calculated. Additionally, bootstrapping was performed based on the calculated haplotypes by generation of 250 bootstrapping replicates. Phased genotypes were then scored by the sum of the scores of their constituent haplotypes, and these values were summed up to score complete haplotype inferences (see Section "Implementation"). The best scored haplotype inference was selected without further optimisation with regard to genotype inference.

Method 3: the analysis described in Method 2 was further refined by an optimisation with regard to genotype inference performed for each alternative most parsimonious set of haplotypes (see Section "Implementation"). 

Method 4: the first haplotype inference was used to optimise the genotype inference. For this purpose, the haplotypes were scored by their frequency in all genotypes of the first haplotype inference. Next, optimisation of the genotypes was carried out as described.

The phased genotypes resulting from all four types of analysis were compared with the corresponding original simulated data set. We first tested whether the original sets of haplotypes, which were used for generating the simulations, could be identified by Method 1, Method 2 and Method 3. Method 4 was left out since different scorings of one haplotype inference do not affect the inferred most parsimonious set of haplotypes. Without added noise, the six original haplotypes could be identified by all three methods. With the addition of noise it was not possible to identify all six original haplotypes in all data sets using the first haplotype inference (Method 1). For three different data sets, this method found fewer than the six original haplotypes (only five out of six were identified in one data set with 6%, 7% and 10% noise). In contrast, all six original haplotypes were correctly inferred by the analyses with bootstrapping (Method 2) and the analyses with bootstrapping and optimisation (Method 3). For all types of analysis, the phased genotypes resulting from each analysis were compared with the original simulated data sets by computing the minimal Hamming distances between an inferred genotype and the corresponding original genotype without noise from the simulation. The minimal Hamming distance was computed as given in Section "Implementation". Based on the minimal Hamming distance, the correctness for all four types of analysis was calculated as follows:

(30) $$100\cdot \frac{600-\text{minimal Hamming distance}}{600-600\cdot 100^{-1}\cdot \text{noise}\%},$$

where 600 is the number of SNP sites in the simulation. The denominator in Formula 30 is motivated by the minimal possible number of incorrectly inferred nucleotides compared to the simulated data without noise (on the right of the sum of the denominator). This value is dependent on the noise used and is subtracted from the number of SNP sites in the data set such that the denominator represents the maximal possible number of correctly inferable nucleotides. The number of correctly inferred nucleotides calculated by the SAT approach is given by the numerator. The mean correctness of the ten different data sets was plotted against the noise (Figure 8).

Without noise, all methods gave predictions close to 100% correctness. With noise added the results of the four analysis methods showed an increasing correctness to the original data in the following order: Method 1 < Method 2 < Method 4 < Method 3. This means that Method 3, which is the method with bootstrapping and genotype optimisation, gave the best results for all values of noise. The comparison between Method 2 and Method 1 demonstrated that the application of bootstrapping in order to select the highest scored haplotype inference (Method 2) gives better results than the method without bootstrapping (Method 1). The distributions of nucleotide distances (minimal Hamming distance) from Method 1 and Method 3 for a given amount of noise were compared by the Kruskal-Wallis test with a significance level of 5%. All p-values except for the 0%-noise case were below 0.05, and consequently the null hypothesis of both distributions being the same was rejected. Although the distributions of nucleotide distances from Method 1 and Method 2 were not significantly different, the mean values of the distances of Method 2 were always smaller than those of Method 1.

### Performance of SATlotyper with unphased SNP data from tetraploid potato genotypes

The performance of SATlotyper was tested using unphased SNP data from the locus *BA213c14t7 *of *Solanum tuberosum*. Locus *BA213c14t7 *corresponds to the sequenced T7-end of the BAC (bacterial artificial chromosome) clone BA213c14 and is located on potato chromosome V between the markers *GP21 *and *GP179 *near the *R1 *gene for resistance to late blight [22] (see Chromosome V in PoMaMo, The Potato Maps and More Database [23,24]). This intergenic sequence region is characterised by high sequence variability. The *BA213c14t7 *sequence also includes SNP sites associated with resistance against the parasitic root cyst nematode *Globodera pallida *[25].

**Figure 10:**
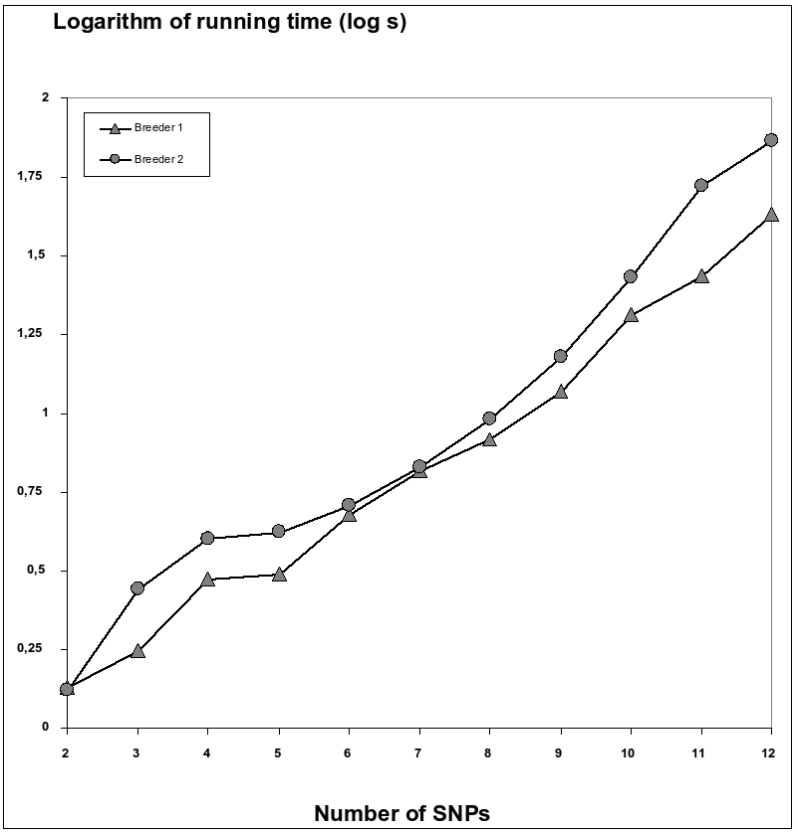
**Performance of SATlotyper with real data sets from tetraploid potato genotypes**. Testing of SATlotyper (Method 1) with two sets of unphased SNP data from the locus *BA213c14t7 *of *Solanum tuberosum *(Figure 9). Analysis of 103 genotypes from breeder 1 and 91 genotypes from breeder 2 took place. The running time was determined when the first haplotype inference was reported. The logarithm of the running time (mean value of ten runs each) was plotted against the number of SNP sites.

As input to SATlotyper, two sets of unphased SNP data of *BA213c14t7 *were generated from 194 heterozygous tetraploid potato individuals from two different breeders: 103 individuals from breeder 1 and 91 individuals from breeder 2. The locus was amplified from genomic DNA of the 194 individuals and the SNP allele dosage (0:4, 1:3, 2:2, 3:1 and 4:0) was estimated for twelve biallelic SNP markers based on the sequence trace files (SNP sites 139, 143, 152, 157, 178, 214, 218, 236, 244, 253, 273, 274; Figure 9; Additional file 2). The resulting unphased SNP data were used as input for a SATlotyper analysis, where only one haplotype inference was calculated (Method 1). The number of SNP sites was varied from two to twelve and the running times were determined. In Figure 10 the log of running time is plotted against the number of SNP sites analysed for the two different data sets (breeder 1, breeder 2). Even for twelve SNP sites, the running time was less than 80 seconds for both data sets. Figure 10 demonstrates that the computational complexity grows exponentially with linear increase of the number of SNP sites.

### Comparison of SATlotyper results with experimentally determined haplotypes

In order to evaluate SATlotyper further, we compared computed haplotypes with experimentally determined haplotypes at the *BA213c14t7 *locus using a subset of nineteen heterozygous tetraploid individuals out of the two populations described above. We identified the haplotypes for twelve SNP sites both computationally and experimentally. The sequence of the *BA213c14t7 *locus and the SNP sites analysed are shown in Figure 9.

#### Computational haplotype inference

The unphased SNP data from the nineteen individuals were used as input for the computational haplotype inference with SATlotyper analysis (Method 2). Up to 250 alternative most parsimonious sets of haplotypes and the corresponding haplotype inferences were calculated. On the basis of the calculated haplotypes bootstrapping was performed (250 samples) in order to score the alternative haplotype inferences. The haplotype inference with the highest score was selected. SATlotyper identified 114 alternative most parsimonious sets of haplotypes for this data set with a minimal number of twelve explaining haplotypes. Additional file 1 (XML output of SATlotyper) gives the input data, the bootstrapping results for all haplotypes and the different scored haplotype inferences which are in order of score. For each alternative haplotype inference the first corresponding genotype inference is given. In Figure 9, the twelve haplotypes obtained from the haplotype inference with the highest bootstrapping score are listed, together with the experimentally determined haplotypes. In addition, an optimisation with regard to genotype inference was performed for all alternative haplotype inferences (Method 3).

#### Experimental haplotype inference

The inference of haplotypes by SATlotyper from experimental SNP data requires the scoring of the SNP allele dosage (zero, one, two, three or four in a tetraploid individual) in PCR amplicons derived from partially heterozygous individuals. Preferential amplification of one allele versus the other may occur at heterozygous loci, resulting in erroneous scores of the allele dosage [25], which leads to the calculation of erroneous haplotypes by SATlotyper. Even with a low percentage of erroneous scores of allele dosage per single SNP site, the combination of errors from several SNP sites can lead to an inflated number of haplotypes that do not exist. To verify haplotype models computed by SATlotyper from experimental SNP data, which are not error free, we performed an independent experimental haplotyping. The number and dosage of haplotypes present at a specific locus in a given individual can be experimentally determined by cloning and sequencing a sufficient number of PCR fragments generated from genomic DNA of that individual at that specific locus. The number of different haplotypes is inferred from the number of consensus sequence variants found in the clone sample, and the haplotype dosage is inferred from the frequency of each consensus sequence variant in the clone sample.

For the subset of nineteen individuals, the haplotypes at the *BA213c14t7 *locus with respect to the twelve SNP sites were determined experimentally (Additional file 2) by the sequencing of at least twenty-four cloned amplicons from each of the nineteen individuals. The number of sequenced clones per tetraploid individual was raised from sixteen, as proposed by Simko [2], to twenty-four per individual, in order to accept as real only haplotypes that could be detected at least twice. This was necessary owing to the possible sequence errors introduced by PCR and Sanger sequencing. In total, 590 amplicon derived clones were sequenced, which revealed ten distinct haplotypes present in the population of nineteen individuals (Table 4, Figure 9). On the basis of the haplotypes observed and the frequency of each haplotype sequence per tetraploid individual, the most likely genotype for each individual was determined (Table 5). The genotype models also allowed determination of haplotype frequencies in the subset of nineteen individuals. Chi-square statistics revealed significant deviation of the observed frequency distribution of haplotype sequences from the numbers expected based on the genotype model in five individuals. Haplotypes H1, H5 and H8 with a frequency of 84% altogether were the most abundant ones (Table 4). The other seven haplotypes had a frequency of less than 5% each. Interestingly, haplotype H4 present in individual S25 shared high similarity (100%, e value: 1^-69^) with the sequence of BAC clone PGEC472P22 originated from the wild potato species *Solanum demissum *[26]. This indicated that haplotype H4 corresponds to an introgression from *Solanum demissum *containing the *R1 *resistance gene [26].

**Table 4:** Experimental haplotypes H1 to H10

Hapl.	SNP139	SNP143	SNP152	SNP157	SNP178	SNP214	SNP218	SNP236	SNP244	SNP253	SNP273	SNP274	Fr. [%]
H1	A	G	T	T	G	C	C	C	C	T	G	T	26.3
H2	A	G	T	T	G	C	A	C	T	C	T	A	1.3
H3	G	G	T	T	G	C	C	C	C	T	G	T	2.6
H4	A	A	T	G	A	C	C	C	T	T	G	A	2.6
H5	G	G	T	T	G	C	A	C	T	C	T	A	19.7
H6	G	G	T	T	G	T	A	C	T	C	T	A	2.6
H7	G	G	A	T	G	C	A	C	T	C	T	A	1.3
H8	G	G	A	T	G	C	C	T	C	T	G	A	38.1
H9	G	G	A	T	G	C	C	C	C	T	G	A	1.3
H10	G	G	T	T	G	C	C	C	C	T	G	A	3.9

Experimental haplotypes for potato locus *BA213c14t7 *resulting from the analysis of nineteen tetraploid individuals (Table 5). SNP sites 139–274 (Figure 9). Values for allele frequency of each haplotype within the nineteen genotypes are based on the model for the most probable genotypes (Table 5) with a maximum of seventy-six possible alleles in this sub-population (100%). Hapl., haplotype. Fr., frequency.

**Table 5:** Haplotypes found in nineteen tetraploid potato individuals and resulting genotype model

Individual	H1	H2	H3	H4	H5	H6	H7	H8	H9	H10	Genotype model	*χ*^2^-value	p-value
S9	19	2			3						1/1/2/5	8.25	0.02
S25				21				27			**4/4/8/8**	0.75	0.39
S50	18		3			3					1/1/3/6	6.00	0.05
S73	18							14			**1/1/8/8**	0.50	0.48
S83	26				2						1/1/1/5	4.76	0.03
S89					7		2	14			**8/8/5/7**	3.26	0.20
S93								26			**8/8/8/8**	0	1
B1					7			17			**8/8/8/5**	0.22	0.64
B4					4			19			**8/8/8/5**	0.71	0.40
B26								24				0	1
B30					79						**5/5/5/5**	0	1
B37	7							16			**8/8/8/1**	0.36	0.55
B43	28		2								1/1/1/3	5.38	0.02
B52	9							14			**1/1/8/8** **1/8/8/8**	1.09 or 2.45	0.30 0.12
B63					2			17	2	8	5/8/9/10	20.79	0.0001
B75					2			9		13	**10/10/5/8**	4.25	0.12
B80	10				12						**1/1/5/5**	0.18	0.67
B86					5			19			**8/8/8/5**	0.22	0.64
B108	20				3						**1/1/1/5**	1.75	0.19

For nineteen individuals, the number of clones obtained per individual for each of the ten haplotypes (Table 4) and the resulting genotype model are shown. For each genotype model, the goodness of fit (chi-square) and the p-values for the deviation of the number of observed clones from the expectation based on the genotype model are calculated. Genotype models which do not significantly deviate from the observed haplotype distribution are shown in bold (significance level: 5%). It is assumed that haplotype frequencies deviate *χ*^2 ^distributed from the genotype model, with # of different alleles -1 degrees of freedom. In the first row of Table 5, the *χ*^2^-value is calculated as follows: $\frac{1}{24}(2{\left(\frac{1}{2}\cdot 24-19\right)}^{2}+4{\left(\frac{1}{4}\cdot 24-2\right)}^{2}+4{\left(\frac{1}{4}\cdot 24-3\right)}^{2})=8.25$. The p-value represents the probability of obtaining haplotype frequencies at least as extreme as the observed ones, given that the genotype model (null hypothesis) is true.

#### Comparison of computed and experimental haplotype and genotype models

From the nineteen individuals analysed, nine haplotypes were identified by both methods (Figure 9). The only experimental haplotype not detected computationally was haplotype H2 (Table 4). H2 was identified only in individual S9, in only two out of twenty-four analysed clones (Table 5). Haplotype H2 did not occur in any of the 114 alternative computed inferences (Additional file 1), which leads to the conclusion that the unphased data do not support haplotype 2 in the most parsimonious set of explaining haplotypes. Three haplotypes were identified computationally but not experimentally. This may result from imperfect input data, for example, from erroneous assignment of SNP allele dosage, which leads to the creation of additional "non real" haplotypes by SATlotyper, which are needed to satisfy the input data. Alternatively, the experimental haplotype inference may have missed rare but real haplotypes owing to underrepresentation of the sequence in the cloned amplicons.

**Figure 11:**
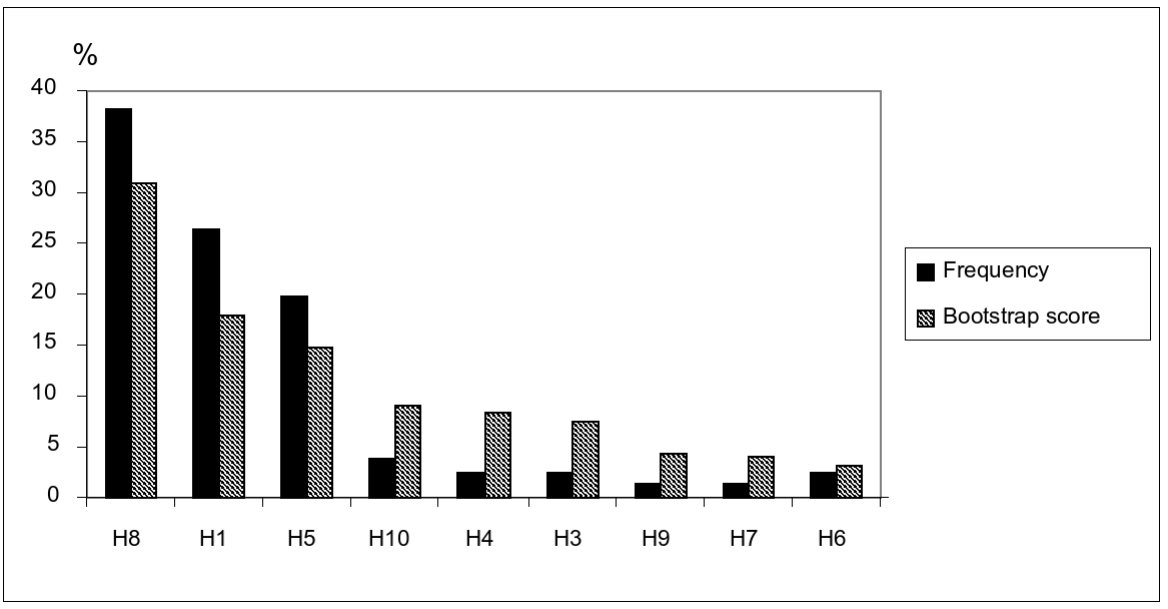
**Comparison of the experimental haplotype frequency with the bootstrapping score of the 9 common haplotypes**. The nine common haplotypes are taken from Figure 9. Haplotype frequency is drawn from Table 4. The bootstrapping score according to Additional file 1 is displayed in percent (100% corresponds to the sum of the bootstrapping scores of the nine common haplotypes). Haplotypes are valued and ordered according to bootstrapping scores.

The high concordance between the experimental results and the prediction is shown in Figure 11. Here, the bootstrapping score is compared with the experimentally determined frequency of the nine common haplotypes.

**Figure 12:**
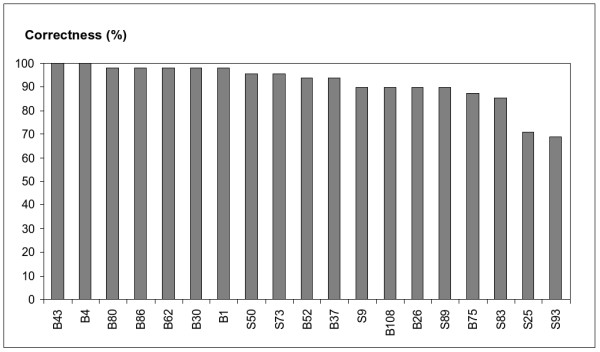
**Correctness of computed phased genotypes compared to the experimental result**. 100% is equivalent with the experimentally determined phased genotype. The correctness was calculated from the Hamming distance, as explained in the text. For individual B52 the experimental genotype model 1/1/8/8 (Table 5) was used for the comparison.

For the nineteen genotypes, we also compared the computed genotype inferences with the experimental ones (Figure 12). The minimal Hamming distance between a predicted genotype and the corresponding experimentally determined genotype was computed as described in Section "Implementation". For every genotype the correctness was derived from the Hamming distance as follows:

(31) $$100\cdot \frac{48-\text{minimal Hamming distance}}{48}.$$

Formula 31 is motivated analogously to Formula 30, except that the amount of noise is not known. As a result, the denominator represents the total number of nucleotides. As shown in Figure 12, we obtained for 80% of the genotypes a correctness of at least 90% when compared with the experimental data. For two individuals (B43, B4) the predicted and experimental haplotypes were exactly the same. For twelve of the nineteen individuals at least three of the four predicted haplotypes were confirmed by the experimental data (Additional file 1, Table 5). The additional optimisation with regard to genotype inference according to Method 3 did not result in a further improvement of the Hamming distance and correctness between predicted and experimental phased genotypes for the nineteen individuals analysed.

## Discussion

Existing approaches for inferring haplotypes from unphased SNP data are only applicable to biallelic and diploid species. This study therefore aimed at the development of an approach for calculating haplotypes in heterozygous polyploid species. Generalising the approach from [7], a Java based program was developed which formulates HIPP for the Boolean satisfiability problem. Instead of giving the constraints for combinatorial sub problems explicitly, SATlotyper generates constraints for summing such that the complexity decreases from exponential to polynomial for polyploid and polyallelic data sets. Other methods for summing based on the SAT approach have been described [17], which are possibly easier to solve by the SAT solver so that a future version of SATlotyper will be further optimised. SATlotyper is able to handle missing SNP information by omitting constraints for such sites so that no unjustified assumptions about nucleotide frequencies have to be made.

For a given data set of unphased genotypes, it is possible by means of SATlotyper to calculate the first most parsimonious set of explaining haplotypes and corresponding phased genotypes (Method 1). One drawback of the parsimony approach is the sparsity of statistical information. A bootstrapping procedure can therefore be used to score haplotype inferences, in case there is more than one possible haplotype inference (Method 2). Since unphased genotypes can also have alternative inferences, it is possible to optimise the phased genotypes in respect to a most parsimonious set of haplotypes and the corresponding bootstrapping scores (Method 3). It is also possible to score the haplotypes without bootstrapping simply by their frequencies in the phased genotypes, which can be used for selecting the best haplotype inference in the case of alternative inferences and for performing an optimisation with regard to alternative genotype inferences (Method 4).

In this study, SATlotyper was tested and evaluated with simulated and experimental data sets of unphased SNP sites from tetraploid individuals. The different SATlotyper methods were compared with the simulated data (Figure 8). Prior to analysis, noise from 0% to 10% was added to the data, to account for erroneous SNP scores in the input data.

Without noise all methods were able to predict the correct set of haplotypes which were used in the simulation (Figure 8). Compared with the original simulation, the haplotype compositions of the phased genotypes were close to the composition of the simulated genotypes (> 99% correctness). With noise added, Method 3 using bootstrapping and optimisation gave the best results (Figure 8). It is likely that the relatively small difference between Method 2 with bootstrapping and Method 1 without bootstrapping (Figure 8) can be explained by the simulation. Because the haplotypes are uniformly distributed, it is very likely that – even in case of noise – all original haplotypes are present in the first found haplotype inference. Thus, an analysis of different distributions of haplotypes in populations is still missing. In the case of real data we would expect a larger difference between the applications of Method 1 and Method 2.

The results obtained when Method 4 (Figure 8) was applied suggest that for some purposes it could be sufficient simply to score the haplotypes corresponding to their frequencies in the phased genotypes for optimising genotype inference. This suggests that data sets that are time consuming to infer can be optimized by Method 4 such that also time consuming bootstrapping can be omitted.

SATlotyper was also applied to an experimental data set of twelve unphased SNP markers, which were scored by sequencing of the amplicons of a 500 bp-region at potato locus *BA213c14t7*. As we have verified only one locus so far, it is not possible to make a firm conclusion how representative the data set of the *BA213c14t7 *locus is. Some variation is expected between different loci with respect to the quality of an amplicon [2] for direct sequencing and whether the amplicon is representative for the genotype at the amplified locus. The performance of the approach was much higher with the experimental data than with simulated data. Nevertheless, the running time increased exponentially with the linearly increasing number of SNP sites (Figure 10).

In addition to performance, the quality of the prediction was evaluated by comparison of predicted haplotypes with experimental haplotypes that were determined by amplicon cloning and sequencing [2]. Unfortunately, the experimental validation of haplotypes is time consuming and expensive. Thus, only a subset of nineteen heterozygous unphased individuals was available for the direct comparison. Furthermore, it has to be taken into account that the evaluation of predicted haplotypes based on comparison with experimentally determined haplotypes is slightly restricted by the fact that the experimental haplotypes are not error-free. In this study, new insights were gained in the experimental set-up for haplotype inference in autotetraploid species by molecular cloning and sequencing of amplicons. In several cases, the observed frequency of amplicon sequences deviated from the expected frequency (0.25, 0.50 or 0.75). One reason could be a difference in the G/C-content of the alleles resulting in altered performances of the PCR-reaction [25,26].

Even slight differences in the initial PCR cycles are enhanced further on in the downstream reactions. This first comparison of computed with experimental haplotypes gave promising results: nine of the ten experimental haplotypes were also identified by SATlotyper prediction out of the sub-population of nineteen individuals (Figure 9). With respect to the phased genotypes, the SATlotyper analysis achieved a correctness of at least 90% (for 80% of the individuals) compared with the experimental result (Figure 12). With the exception of Method 1, all SATlotyper methods gave similar results with this data set.

## Conclusion

The study demonstrates that HIPP can efficiently be solved for data sets of unphased SNP sites from heterozygous polyploids by a generalisation of the SAT approach from [7]. Our results are encouraging for the future application and further development of SATlotyper. Existing or newly generated unphased SNP data can be analysed by SATlotyper to infer haplotypes. Haplotype information can be used instead of individual SNP sites in association mapping that exploits the biodiversity in existing cultivars and breeding lines [2]. Compared with methods based on individual SNP sites, the haplotype mapping method significantly improves the power and robustness of gene mapping techniques [27] as there are fewer haplotypes than SNP sites [2].

## Availability and requirements

SATlotyper was developed in the scope of GABI (Genome analysis of the plant biological system) projects and can be downloaded from the SATlotyper project page [28] of GabiPD, The GABI Primary Database [29]. The software is distributed as a Java JAR file and requires Java Runtime Environment 1.5.0 or higher. For the user's convenience, the downloadable archive contains statically linked versions of MiniSat [11,12] and MiraXT [13]. The software is accessed from command line. Under UNIX like systems the program runs out of the box with MiniSat [11,12], MiraXT [13] and the Sat4J solver [14]. Users with Microsoft^® ^Windows are restricted on running the Sat4J solver. SATlotyper is freeware for scientific use and is distributed under the SATlotyper licence, which is also included in the downloadable package.

## Authors' contributions

JN developed and implemented SATlotyper, performed all computational haplotype inferences and wrote most of the paper together with BK. GG performed the experimental haplotyping and participated in writing. RB contributed to the design and the implementation of SATlotyper. SD and CG initiated the prediction of haplotypes for potato and provided ideas. UA generated the SNP data and initially supervised the experimental part of the study together with CG, who also provided ideas for the development of SATlotyper and participated in writing. JS initiated the development of SATlotyper based on the SAT approach, he supervised and co-ordinated with BK the computational part of the study. BK also compared the results of computational and experimental haplotypings and co-ordinated the study. All authors read, edited and approved the final manuscript.

## Supplementary Material

Additional file 1 **Computational haplotype inference – XML output of SATlotyper**. The XML output of SATlotyper is based on the treelike structure of the result. The root of the tree is marked by the <*result*> tag. The next level in the tree consists of the original input data (<*source*>), the scored haplotype (<*haplotype*>) list derived by bootstrapping (<*bootstrapping*>) and the calculated alternative inferences of the unphased input data (<*haplotypings*>). The haplotypings branch contains at least one inference of the original input data (<*haplotyping*>) with the corresponding most parsimonious set of haplotypes (<*haplotypes*>) and phased genotypes (<*genotypes*>) as subelements where a genotype (<*genotype*>) contains the ploidy-specific number of inferred haplotypes. Click here for file

Additional file 2 **Experimental methods**. This file describes the experimental methods used for (i) the generation of unphased SNP data from tetraploid potato individuals and (ii) the analysis of haplotype inference by amplicon cloning and sequencing. Click here for file
