# Supplementary material for: Haplotype inference from unphased SNP data in heterozygous polyploids based on SAT
Source: BMC Genomics. 2008 Jul 30;9:356. doi: 10.1186/1471-2164-9-356 (PMC2566320; doi:10.1186/1471-2164-9-356)
Supplement: Additional file 2 — Experimental methods. This file describes the experimental methods used for (i) the generation of unphased SNP data from tetraploid potato individuals and (ii) the analysis of haplotype inference by amplicon cloning and sequencing. [file 1471-2164-9-356-S2.pdf]

Experimental methods

Generation of unphased SNP data from tetraploid potato individuals

50 ng genomic DNA each of 194 tetraploid, heterozygous potato individuals (kindly provided by the potato breeding companies Saka-Ragis, Windeby, Germany, and Böhm-Nordkartoffel Agrarproduktion, Ebstorf, Germany) were amplified with primers specific for the T7 end of potato BAC clone BA213c14 (forward: 5'-CAATTGATTCATTTTATGTAGCGAG-3', reverse: 5'-TCTTGACGCAAACCTCTGCGAG-3') according to Rickert *et al.* [1]. The *BA213c14t7* amplicons of 650 base pairs were purified by treatment with ExoSAP-IT® (USB, Cleveland, USA) according to supplier's instructions, and were sequenced at the MPIZ DNA core facility using Applied Biosystems Abi Prism 3730 sequencer and BigDye-terminator v3.1 chemistry. The premixed reagents were from Applied Biosystems (Weiterstadt, Germany). The forward primer was used as sequencing primer. SNPs were detected in the sequence trace files as overlapping base calling peaks. SNP allele dosage in heterozygous tetraploid individuals (0:4, 1:3, 2:2, 3:1 or 4:0) was estimated from the height ratio of the overlapping base calling peaks manually and using the Data Acquisition and Analysis Software DAx (van Mierlo Software Consultancy, www.dax.nl).

Experimental haplotype inference

BA213c14t7 amplicons generated from 19 of the 194 tetraploid potato individuals were ligated in the pGEM-T vector (Promega, Madison, WI, USA) according to supplier's instructions, and were transformed into E. coli strain DH5α using standard protocols. From 24 to 88 independent bacterial colonies per individual, the *BA213c14t7* insertion was amplified and sequenced as described above. Sequence variants occurring at least twice in the sample of sequenced amplicons per individual were considered as true haplotypes.

References

1. Rickert AM, Kim JH, Meyer S, Nagel A, Ballvora A, Oefner PJ, Gebhardt C: **First-generation SNP/InDel markers tagging loci for pathogen resistance in the potato genome**. *Plant Biotechnology Journal* 2003, **1**(6):399–410.
